# Semi-supervised GAN with hybrid regularization and evolutionary hyperparameter tuning for accurate melanoma detection

**DOI:** 10.1038/s41598-025-17756-x

**Published:** 2025-08-30

**Authors:** Alireza Golkarieh, Parsa Razmara, Ahmadreza Lagzian, Amirhosein Dolatabadi, Seyed Jalaleddin Mousavirad

**Affiliations:** 1 Department of Mechanical Engineering, University of Michigan, Michigan, USA; 2 University of Southern California, Los Angeles, CA USA; 3 The city university of New York, New York, NY USA; 4 Department of Biomedical Engineering, Science and Research Branch, Islamic Azad University, Tehran, Iran; 5 Department of Computer and Electrical Engineering, Mid Sweden University, Sundsvall, Sweden

**Keywords:** Melanoma detection, Generative adversarial network, Semi-supervised learning, Hyperparameter optimization, Artificial bee colony, Cancer, Electrical and electronic engineering

## Abstract

Melanoma, influenced by changes in deoxyribonucleic acid (DNA), requires early detection for effective treatment. Traditional melanoma research often employs supervised learning methods, which necessitate large, labeled datasets and are sensitive to hyperparameter settings. This paper presents a diagnostic model for melanoma, utilizing a semi-supervised generative adversarial network (SS-GAN) to enhance the accuracy of the classifier. The model is further optimized through an enhanced artificial bee colony (ABC) algorithm for hyperparameter tuning. Conventional SS-GANs face challenges such as mode collapse, weak modeling of global dependencies, poor generalization to unlabeled data, and unreliable pseudo-labels. To address these issues, we propose four improvements. First, we add a reconstruction loss in the generator to minimize mode collapse and maintain structural integrity. Second, we introduce self-attention in both the generator and the discriminator to model long-range dependencies and enrich features. Third, we apply consistency regularization on the discriminator to stabilize predictions on augmented samples. Fourth, we use pseudo-labeling that leverages only confident predictions on unlabeled data for supervised training in the discriminator. To reduce dependence on hyperparameter choices, the Random Key method is applied, enhanced through a mutual learning-based ABC (ML-ABC) optimization. We evaluated the model on four datasets: International Skin Imaging Collaboration 2020 (ISIC-2020), Human Against Machine’s 10,000 images (HAM10000), Pedro Hispano Hospital (PH2), and DermNet datasets. The model demonstrated a strong ability to distinguish between melanoma and non-melanoma images, achieving F-measures of 92.769%, 93.376%, 90.629%, and 92.617%, respectively. This approach enhances melanoma image classification under limited labeled data, as validated on multiple benchmark datasets. Code is publicly available at https://github.com/AmirhoseinDolatabadi/Melanoma.

## Introduction

According to the World Health Organization (WHO), skin cancer is a significant global health issue, with over a million new cases diagnosed each year. This accounts for approximately one-third of all cancer diagnoses. The primary cause of skin cancer is ultraviolet (UV) radiation from the sun. Therefore, the WHO recommends that individuals with fair skin limit sun exposure and regularly inspect their skin for unusual growths or markings, as noted by the research of Hu et al.^1^.

Skin cancer is generally classified into two categories: melanoma and non-melanoma, as reported by Weir et al.^2^. According to the Global Cancer Observatory 2020 (GLOBOCAN 2020) report, there were 324,635 new cases of melanoma and 1,198,073 cases of non-melanoma skin cancer in 2020. Melanoma resulted in 57,043 deaths, while non-melanoma was responsible for 63,731 deaths, as noted by Bray et al.^3^. The age-standardized rate (ASR) for melanoma was 3.4 per 100,000 individuals, in contrast to 11 per 100,000 for non-melanoma. Ferlay et al.^4^ further noted that the age-standardized mortality rates were 0.56 per 100,000 for melanoma and 0.6 per 100,000 for non-melanoma. Early detection is critical, as it can significantly reduce the risk of death from skin cancer. For example, a study in Pennsylvania, United States of America (USA), reported the detection of many early-stage melanomas. These melanomas were initially superficial lesions but could progress into more dangerous forms without timely intervention. In recent years, image analysis technologies have played an increasing role in supporting early diagnosis. According to Liu and Kawashima^5^, artificial intelligence (AI)-assisted tools have emerged as valuable aids, providing faster and often more accurate assessments of skin lesions compared to manual inspections by clinicians.

### Research gaps

Numerous investigations into melanoma have predominantly employed supervised learning methods, as seen in studies by Jayaraman et al.^6^, Poornima et al.^7^, and Luu et al.^8^. This technique involves training algorithms on categorized skin image datasets, where images are distinctly labeled as either melanoma or non-melanoma. The algorithm develops the ability to detect melanoma-specific patterns and features, enhancing its diagnostic precision for novel images. Nonetheless, these methods face substantial challenges. They require extensive, accurately labeled datasets, which are often scarce in the medical sector due to confidentiality concerns and the variable nature of clinical scenarios. Supervised learning models are also susceptible to overfitting if trained on limited or narrowly specific datasets, which can reduce their efficacy in unfamiliar cases. To mitigate these challenges, semi-supervised approaches, such as SS-GANs, offer a promising alternative. SS-GANs utilize a smaller set of labeled data combined with a larger pool of unlabeled data. This approach significantly enhances the accuracy and resilience of the learning process, as noted by Xu et al.^9^. Although SS-GANs provide important benefits over traditional supervised methods by leveraging both labeled and unlabeled data, they still suffer from several fundamental issues. These limitations significantly affect their performance in clinical environments.

One of the major limitations of SS-GANs is mode collapse, a phenomenon where the generator produces limited or repetitive outputs rather than covering the full data distribution, as recorded by Tomar and Gupta^10^. In melanoma detection, this becomes particularly problematic. Synthetic lesion images generated under mode collapse often lack the variety of visual characteristics found in real clinical data. These characteristics include differences in shape, color gradation, size, and irregularities at the borders. As a result, the model becomes biased toward a narrow range of lesion types, ignoring atypical or rare presentations. This significantly reduces generalizability, resulting in poor detection rates for rare melanoma subtypes. In clinical contexts, where early and accurate diagnosis is critical, failure to capture lesion heterogeneity poses a serious risk.

Another key problem is the inability of conventional SS-GANs to model long-range dependencies in medical images, as confirmed by Pacal et al.^11^. This is particularly concerning in dermoscopic image analysis. Accurate melanoma diagnosis depends not only on localized features but also on broader structural patterns across the lesion. For instance, asymmetrical pigment distribution, irregular streaks, and border asymmetries span across the lesion. These characteristics require a large receptive field to be interpreted correctly. Standard convolutional architectures often overlook these relationships, resulting in shallow feature representations. In practical terms, this limitation means the model may overlook subtle visual cues that are critical for identifying malignant lesions. This reduces diagnostic accuracy in real-world clinical scenarios.

SS-GANs also struggle with poor generalization to unlabeled data, which is abundant in medical domains as noted by Khan et al.^12^. In real-world applications, particularly for melanoma, labeled datasets are limited due to the need for expert annotation and privacy constraints. When only a small fraction of samples is labeled, the model relies heavily on the discriminator. It uses the discriminator to learn meaningful decision boundaries. However, if the discriminator fails to differentiate between malignant and benign features with limited guidance, its predictions on unlabeled samples become unreliable. This is particularly problematic for ambiguous or borderline lesions that deviate from textbook appearances. As a result, the model may misclassify cases. This reduces its effectiveness in assisting clinicians and undermines trust in AI-assisted diagnosis.

A further limitation involves the use of unreliable pseudo-labeling strategies, as demonstrated in the study by Peng et al.^13^. In many SS-GAN implementations, unlabeled data are automatically assigned labels based on the discriminator predictions. Without proper confidence filtering, this process can introduce substantial noise into the training pipeline. For example, if low-confidence samples are mistakenly labeled as melanoma or non-melanoma, the model learns incorrect patterns. This may lead to benign lesions being misinterpreted as having malignant features or vice versa. Such misclassifications in a clinical setting are dangerous. False positives may lead to unnecessary biopsies, while false negatives can result in missed diagnoses. Therefore, ensuring the quality of pseudo-labels is vital for maintaining diagnostic integrity and patient safety.

Beyond these architectural issues, there are also important optimization challenges that affect model performance and usability. Determining the most effective hyperparameters for melanoma detection models is complex due to variations in skin characteristics and imaging conditions across different datasets. This challenge requires sophisticated tuning techniques. Traditional methods such as grid search and genetic algorithms are frequently employed. Grid search evaluates every combination of hyperparameters within a set range to identify the optimal configuration, as used in the study by Wu et al.^14^. Conversely, genetic algorithms utilize principles from evolutionary biology to evolve hyperparameters towards the best solutions. Nonetheless, grid search can be prohibitively resource-demanding and inefficient as it exhaustively explores all possible combinations. Meanwhile, genetic algorithms struggle with consistent convergence and may prematurely settle on suboptimal solutions, as reported by Jiang et al.^15^.

### Hypothesis

To address key challenges in SS-GANs for melanoma detection and the complexity of hyperparameter optimization, this study proposes five targeted hypotheses:

#### • hypothesis 1

• Integrating a reconstruction loss into the generator objective function is expected to mitigate mode collapse. This modification encourages the generation of structurally consistent and diverse synthetic lesion images. It is expected to improve subtype coverage and enhance generalizability across various patient cases.

#### • hypothesis 2

• Employing self-attention mechanisms in both the generator and the discriminator is hypothesized to strengthen the modeling of long-range spatial dependencies in dermoscopic images. This design enables the network to capture global contextual patterns, such as asymmetric pigmentation and irregular structures, allowing for more accurate predictions.

#### • hypothesis 3

• Incorporating consistency regularization into the discriminator will help enforce prediction stability across multiple augmented versions of the same input. This approach is expected to reduce sensitivity to noise and improve generalization, particularly on unlabeled data from imbalanced or heterogeneous datasets.

#### • hypothesis 4

• Utilizing a confidence-based pseudo-labeling strategy will enhance the reliability of training signals from unlabeled data. By selecting only high-confidence samples for supervised loss, this strategy aims to minimize label noise and stabilize training, ultimately improving diagnostic accuracy in clinical settings.

#### • hypothesis 5

• Enhancing the ABC algorithm with a mutual learning mechanism will improve hyperparameter optimization. This mechanism fosters collaborative exploration among candidate solutions, helping overcome the limitations of traditional tuning methods such as grid search and standard ABC. Consequently, the model should achieve higher diagnostic accuracy. This holds true, even under heterogeneous imaging conditions and varied skin characteristics.

Together, these five hypotheses form a comprehensive framework for addressing the core limitations of conventional SS-GANs and optimizing performance, specifically tailored to the demands of melanoma image analysis.

### proposed approach overview

This study presents an advanced model for melanoma diagnosis that integrates an SS-GAN with an improved ABC algorithm for hyperparameter optimization. The SS-GAN framework utilizes a discriminator to categorize samples into three classes: melanoma (positive), non-melanoma (negative), and real versus fake.

To enhance the robustness and diagnostic reliability of the model, several architectural improvements are introduced. A reconstruction loss is incorporated into the generator to reduce mode collapse and maintain diversity among generated lesions. Self-attention modules are added to both the generator and the discriminator to enhance global context modeling. This capability is crucial for identifying intricate dermoscopic patterns. Additionally, consistency regularization is applied to the discriminator to stabilize the prediction outputs across augmented inputs. A confidence-based pseudo-labeling mechanism is also used. It selectively includes only high-confidence unlabeled samples to reduce training noise and improve label quality. To reduce dependency on fixed hyperparameter settings, the model utilizes the Random Key encoding method. This is further enhanced with a mutual learning-based ABC algorithm. This algorithm promotes a dynamic balance between exploration and exploitation during the optimization process.

### key contributions

The main contributions of this paper are outlined as follows:

- This paper implements an SS-GAN for melanoma detection, offering a substantial contribution to the field. This method effectively addresses the limited availability of labeled data, a common challenge in medical imaging. The model uses a semi-supervised learning (SSL) framework. It combines both labeled and unlabeled data to enhance the learning process. This makes the approach particularly useful when acquiring fully labeled datasets is difficult or costly. As a result, the model achieves notable gains in classification performance on benchmark melanoma datasets, validating its capability to operate effectively with scarce labeled data.
- To overcome the limitations of conventional SS-GANs, the model integrates four architectural enhancements. (a) A reconstruction loss in the generator prevents mode collapse and maintains lesion diversity. (b) Self-attention mechanisms in both the generator and discriminator capture long-range dependencies in dermoscopic images. (c) Consistency regularization on the discriminator stabilizes predictions under variations in augmented inputs. (d) A confidence-guided pseudo-labeling mechanism reduces label noise and improves learning from unlabeled samples. These improvements enable the model to capture the complexity of melanoma features more accurately and enhance its generalization across diverse clinical cases.
- Hyperparameter tuning plays a crucial role in achieving optimal performance, particularly in complex models such as GANs. In this study, the ABC algorithm is used to dynamically and efficiently adjust the hyperparameters of the model. This bio-inspired approach eliminates the need for exhaustive search. It reduces computational costs and enhances the scalability of the model for deployment in real-world clinical environments.
- A key innovation is the enhancement of the ABC algorithm through a mutual learning mechanism. In this improved framework, candidate solutions within the ABC population share information during the search process. This cooperation accelerates convergence and helps avoid getting stuck in local optima. The algorithm promotes collaborative exploration of the hyperparameter space. This process identifies more effective configurations, which significantly improve the performance of the model in melanoma diagnosis.

### Organization of the paper

The remainder of this study is organized as follows: Sect. 2 reviews previous research in melanoma detection. Section 3 describes our proposed method for diagnosing melanoma, while Sect. 4 presents the results of our experiments. Finally, Sect. 5 provides a conclusion for this paper.

## Related work

In recent years, a range of techniques for diagnosing melanoma has emerged, including approaches based on machine learning (ML), deep learning (DL), and transfer learning (TL). Each of these methodologies is explored in detail in the following sections of this paper.

### Machine learning

Over the years, the techniques for automatically classifying melanoma have evolved substantially, particularly in the early 2000s, a crucial period for introducing these methods. In these foundational years, there was an emphasis on using simple, manually crafted features to distinguish between melanoma and non-melanoma cases. These essential features focused on vital characteristics such as shape, color, and texture. Detailed studies investigated shape-related attributes^16^ color details^17^ and textural properties^18^ as primary factors for differentiation^19^.

Several studies employed supervised machine learning with explicit feature extraction followed by conventional classifiers. In 2023, Poornima et al.^7^ worked with the MedNode dataset. They applied image enhancement and segmentation to extract features related to ABCD (asymmetry, border, color, diameter) and statistical attributes. Classification was conducted using feed-forward neural networks (FFNN), support vector machine (SVM), and k-nearest neighbors (KNN). Luu et al.^8^ analyzed the optical properties of skin using Mueller matrix-based imaging. These polarimetric data were processed using KNN, decision trees (DT), and SVM for cancer detection. Naghavipour et al.^20^ offered an open-source guide to ML for melanoma recognition in dermoscopy. It covers data processing, lesion boundary detection, and model comparison among artificial neural network (ANN), SVM, and KNN. Their findings indicate that ANN achieves superior accuracy. In 2024, Camargo et al.^21^ presented a skin lesion classification framework combining KNN and SVM models. Their method incorporated image preprocessing via Otsu thresholding and feature extraction based on the criteria of ABCD.

Some studies used hybrid methods or techniques enhanced by metaheuristics to improve performance. In 2023, Liu and Kawashima^5^ introduced a method for melanoma identification. It uses reinforcement learning for skin segmentation and applies an enhanced fish migration optimizer (EFMO) for both feature extraction and SVM tuning. Ghahfarrokhi et al.^22^ developed a computer-aided diagnosis (CAD) framework. It combines an online region-based active contour model (ORACM) for region of interest (ROI) detection with a non-dominated sorting genetic algorithm II (NSGA-II) for feature selection. They utilized a pattern recognition neural network (PatNet) for classification, achieving strong performance. Chaugule et al.^23^ proposed an automated tool for skin cancer diagnosis. Their pipeline includes image preprocessing, feature extraction, and classification via convolutional neural networks (CNNs), SVMs, and random forests (RF). Alenezi et al.^24^ built a hybrid system by integrating deep residual networks with Relief-based feature selection. Model performance was further refined using SVM and Bayesian optimization. In 2025, Paramasivam et al.^25^ utilized the Visual Geometry Group 16 (VGG16) DL architecture to extract relevant features from skin lesion images. They applied several ML classifiers, including DT, KNN, logistic regression (LR), RF, and SVM, for lesion classification. The bat algorithm (BA) was employed to optimize model hyperparameters.

Some studies explored unsupervised learning and clustering techniques. In 2024, Romero-Morelos et al.^26^ introduced a non-invasive diagnostic strategy for melanoma by analyzing the fractal characteristics of skin lesion boundaries. They applied principal component analysis (PCA) and iterative k-means clustering, two unsupervised learning methods, to perform classification.

Several ensemble and fusion-based models were also proposed. In 2024, Courtenay et al.^27^ investigated the use of near-infrared hyperspectral imaging for identifying non-melanoma skin cancers. Their method utilized a hybrid CNN to extract features and applied SVM for classification. They also evaluated how transfer learning influences model performance. In 2025, Mishra et al.^28^ proposed a novel melanoma identification method that combines multiple KNN algorithms into a fused model. This model is further optimized using an indoor positioning system (IPS) to enhance classification accuracy.

### Deep learning

While classical machine learning methods laid the foundation for melanoma classification, the field has progressively moved toward more data-driven deep learning approaches that can automatically extract high-level features.

Many studies focus on supervised models that use CNNs and labeled datasets to learn useful features. In 2023, Tembhurne et al.^29^ proposed an integrated skin cancer detection pipeline that combines CNNs for feature extraction with traditional ML techniques for feature refinement. Their method incorporated contourlet transforms and local binary pattern (LBP) histograms to enhance image interpretation. Waheed et al.^30^ developed a graphics processing unit (GPU)-accelerated DL system. This method utilized a pre-trained CNN to differentiate melanoma from benign lesions. Bandy et al.^31^ developed a CNN-based classification model designed to enhance the receiver operating characteristic–area under the curve (ROC-AUC) scores. They integrated various AI clustering algorithms to enhance decision-making. In 2024, Angeline et al.^32^ introduced a computer-aided skin cancer detection system that employs a two-phase CNN to extract high-level features. These features are then combined with ABCD dermatological rules and ensemble classifiers, such as gradient boosting and extreme gradient boosting (XGBoost), to enhance the reliability of classification. Veeramani et al.^33^ proposed a double-decker CNN (DDCNN) for melanoma classification. It incorporates a novel ‘F’ Flag feature, intra-class variance score, and hybrid feature fusion to improve the diagnosis of malignant lesions. Veeramani et al.^34^ suggested a multi-class skin cancer detection system. It combines you only look once version 7 (YOLOv7) with explainable AI (XAI) techniques, such as local interpretable model-agnostic explanations (LIME) and Shapley Additive exPlanations (SHAP). This system ensures transparent and reliable melanoma classification across eight skin cancer types, including melanoma. In 2025, Hu and Zhang^35^ introduced a melanoma recognition framework that incorporates multiple CNNs, with an ML-ABC algorithm guiding the optimization. The model also incorporates reinforcement learning strategies to more effectively address class imbalance issues during training. Kaur et al.^36^ proposed an end-to-end CAD solution for melanoma. Their framework begins with image preprocessing using morphological filters and employs context-aware deep aggregation networks for feature extraction. It then applies deep learning-based segmentation and classification to distinguish between benign and malignant skin lesions. Adamu et al.^37^ developed an innovative approach for tuning CNN hyperparameters in skin cancer analysis, relying on the manta ray foraging optimization (MRFO) algorithm. This method systematically refines model parameters, yielding enhanced classification accuracy.

Another area of research explores semi-supervised and self-supervised learning to address the challenge of small or partially labeled datasets. In 2023, Dong et al.^38^ proposed a semi-supervised multi-path grid network (SSGNet) that integrates colorization and classification branches. These branches share an encoder and utilize spatial self-attention, along with a multi-scale residual grid structure, to enhance feature extraction. Zhou et al.^39^ developed an SSL method. This method combines label smoothing with consistency regularization. The goal is to reduce the impact of noisy pseudo-labels and improve classification reliability in medical imaging. Zhu et al.^40^ introduced a semi-supervised framework that incorporates two components: noisy-consistent sample learning (NCSL) and uncertainty-aware attention. These components help refine pseudo-labels and integrate discriminative features for improved skin lesion classification. Peng et al.^13^ proposed a dynamic semi-supervised approach. The model combines multi-curriculum pseudo-labeling (MCPL) with label smoothing (LS). It adjusts class-wise confidence thresholds to enhance learning from imbalanced and unlabeled datasets, including those containing melanoma images. In 2024, Lal et al.^41^ presented a semi-automated framework using self-supervised learning. The method utilizes deep convolutional GANs (DCGAN) to augment the training data, starting with unlabeled images and refining the classification using labeled ones. Yuan et al.^42^ developed a semi-supervised melanoma classifier using self-feedback threshold focal learning (STFL). This method dynamically selects unlabeled samples and applies focal loss to handle class imbalance during training. In 2025, Wang^43^ proposed a self-supervised learning approach for multimodal, multilabel skin lesion classification, utilizing clustering-based pseudo-labels and a label-relation-aware module with attention-enhanced CNNs.

Some newer models utilize GANs and transformer architectures to enhance data variation and feature extraction. Albraika et al.^44^ designed a DL-based diagnostic model for melanoma. The process began with image enhancement and augmentation. Segmentation was then performed using the k-means clustering algorithm. Features were extracted using capsule networks, and classification was performed using sparse autoencoders (SAE) combined with crow search optimization (CSO). Sukanya et al.^45^ introduced a multi-stage framework. First, the self-adaptive sea lion optimization algorithm was used to segment the lesion. Then, texture features were analyzed using deep belief networks (DBNs) for classification. Nirmala et al.^46^ proposed a method combining GAN-generated synthetic melanoma images with a CNN model. The model uses the VGG-16 architecture for efficient skin lesion classification. This method reduces preprocessing time and enhances diagnostic accuracy. Wang et al.^47^ proposed a two-step detection system. In the first phase, they applied styleGANs with adaptive discriminator augmentation to increase dataset diversity. In the second phase, they used a dual-branch vision transformer to extract deep features for melanoma detection. Ju et al.^48^ built a DL-based identification model integrating dilated convolutions with off-policy proximal policy optimization (PPO). They also utilized a GAN to generate synthetic samples, which helped to resolve the class imbalance. Suryanarayana et al.^49^ employed a spatiotemporal joint graph convolutional network (STJGCN) combined with a red panda optimization algorithm (RPOA) for classification. Noise reduction was achieved through preprocessing using the information exchange multi-Bernoulli filter (IEMBF). Veeramani et al.^50^ submitted a melanoma information improved generative adversarial network (MELIIGAN) framework. It uses AI-based reconstruction, stacked residual blocks, and hybrid loss functions to aid early melanoma diagnosis from dermoscopic images.

### Transfer learning

Due to the limited dataset sizes, many recent studies employ transfer learning by applying pre-trained models to enhance melanoma detection. These methods utilize information learned from large, labeled datasets to enhance classification, particularly in cases where domain-specific data is limited.

A major group of studies relies on standard deep feature extraction with pre-trained CNNs. These methods utilize well-established architectures for image representation, which precede classification. In 2023, Thanka et al.^51^ designed an integrated skin cancer recognition framework that leverages VGG16 for extracting visual patterns and combining them with the XGBoost classifier to enhance prediction reliability. Shobarani et al.^52^ applied deep transfer learning for melanoma classification. Their approach involved enhancing images using filters and histogram equalization. Then, they utilized a densely connected convolutional network with 121 layers (DenseNet-121), the Inception architecture with a residual network version 2 (Inception-Resnet-V2), and the extreme Inception (Xception) architecture for classification. Koppolu et al.^53^ employed the efficient convolutional neural network–baseline model B0 (EfficientNetB0) architecture, renowned for its computational efficiency, in conjunction with data augmentation strategies to categorize skin lesions using the HAM10000 dataset, with a focus on detecting melanoma. Orhan and Yavşan^54^ implemented a melanoma detection approach driven by AI using popular deep learning models such as Alex Krizhevsky’s convolutional neural network (AlexNet) and mobile network (MobileNet). Their model was trained on a large image dataset and envisioned integration into a smartphone application for early screening. In 2025, Shakya et al.^55^ assessed three deep-learning approaches for melanoma detection. They utilized transfer learning on VGG-19, ResNet-18, and MobileNet, paired with classifiers such as DTs and SVMs. The pipeline also involves preprocessing methods, such as resizing and noise removal.

Another category comprises transfer learning methods that either utilize multiple models in conjunction or integrate deep learning with traditional machine learning techniques. In 2023, Hussain et al.^56^ introduced a novel dataset called Nailmelanoma, tailored for nail melanoma analysis. They evaluated seven different CNNs and applied model compression techniques to support deployment on mobile platforms, enabling the development of swift and lightweight diagnostic solutions. Kalyani et al.^57^ developed a hybrid model that combines the arithmetic optimization algorithm with an ensemble deep learning approach. It includes Gabor filtering for feature extraction and a U-shaped network (U-Net) for segmentation and integrates multiple deep networks for a detailed melanoma diagnosis. Viknesh et al.^58^ proposed a diagnostic model that merges deep learning with SVMs. The system uses CNNs for feature learning and SVM with radial basis function (RBF) kernel for classification, targeting both web and mobile platforms. In 2024, Meswal et al.^59^ introduced a novel melanoma identification method based on a weighted ensemble strategy, which integrates predictions from four pre-trained deep learning models: InceptionV3, VGG16, Xception, and ResNet50. The final decision is derived by assigning weights to each model based on its classification performance, thereby enhancing diagnostic precision.

A smaller group incorporates optimization and interpretability techniques into transfer learning frameworks. In 2023, Pérez and Ventura^60^ created a CNN-based system that operates within an active learning framework. The model iteratively improves through expert input and batch query selection, resulting in strong generalization across various datasets. In 2025, Verma et al.^61^ proposed a full diagnostic pipeline built on a dual-branch CNN-transformer combination. This system performs joint feature extraction and segmentation. Convolutional network next (ConvNeXt) is used with attention-based fusion, while ResNet-50 is refined with gradient-weighted class activation mapping (Grad-CAM) to enhance interpretability. They applied Henry gas solubility optimization (HGSO) and water strider algorithm (WSA) metaheuristics to further optimize performance.

A few studies have adopted augmentation-enhanced transfer learning, where augmented data improves model training despite the limited availability of labeled samples. In 2024, Vishnu et al.^62^ developed a detection framework that combines two neural networks trained on augmented dermoscopic data. This setup helps compensate for the limited number of samples, enabling the classification of lesions into six categories with greater robustness. Bazgir et al.^63^ developed a CNN architecture for skin disease diagnosis with a focus on melanoma. Their approach optimizes an Inception-based network using preprocessing filters to improve input clarity. The model is trained on a dataset of 2637 curated dermoscopic images.

**Table 1:** Comparative overview of ML, DL, and TL techniques for melanoma detection.

Reference	Method description		Key Innovation	Dataset	Accuracy	Limitation
Poornima et al.^7^		Images refined through enhancement and segmentation, analyzed using FFNN, SVM, and KNN	Blends traditional techniques and ML for stable analysis	MedNode	0.967	Results are influenced by starting image clarity
Luu et al.^8^		The Mueller matrix technique was employed to capture skin optical traits, then assessed with ML algorithms	Combines Mueller-based optics with ML to enrich insights	MedNode	0.94	Demands an accurate setup of optical devices
Naghavipour et al.^20^		Educational guide demonstrating how to detect melanoma using ML workflows	Offers step-by-step training content for learners	PH2	0.835	Learning results vary with user interaction level
Camargo et al.^21^		ABCD rule-based features extracted from lesions and classified with KNN and SVM after Otsu segmentation	Applies hybrid ML models for skin lesion evaluation	ISIC	0.6409	Outcomes affected by preprocessing accuracy
Liu and Kawashima^5^		Reinforcement learning was applied to segment skin images, and EFMO was utilized for extracting diagnostic features	Refines SVM using EFMO and boosts segmentation performance	ISIC	0.85	Performance hinges on EFMO optimization quality
Ghahfarrokhi et al.^22^		ORACM used to outline ROI; features refined by NSGA II and classified with c	Improves ROI extraction with optimized feature selection	PH2	0.9312	Difficulty handling datasets with wide variability
Chaugule et al.^23^		Fully automated detection using preprocessing stages and ML-based classification strategies	End-to-end detection from preprocessing through classification	ISIC 2019	0.856	Intensive processing power may be necessary
Alenezi et al.^24^		Combined use of SVM with Bayesian optimization for parameter selection	Advances feature quality and refines the tuning process	ISIC 2019	0.9862	Risk of overfitting due to data-specific traits
Paramasivam et al.^25^		VGG16 as base extractor, followed by ML classification models fine-tuned using BA	Derives features with VGG16 and fine-tunes via BA	ISIC	0.8115	Requires a BA for tuning efficiency
Romero-Morelos et al.^26^		System estimates lesion fractality, then applies PCA and iterated k-means for categorization	Applies a non-contact approach with fractal metrics and clustering	ISIC	0.724	Effectiveness is limited to fractal feature reliance
Courtenay et al.^27^		Near-infrared imaging paired with hybrid CNNs and SVM to identify non-melanoma cancers	Leverages hyperspectral inputs and uses transfer learning	DETTECTHIA	0.85	Needs access to specialized imaging tools
Mishra et al.^28^		Fused-KNN model within an IPS framework to improve image quality and classification	Consolidates KNN variants to enhance classification precision	ISIC 2020	0.9445	The classification depends on precise KNN tuning
Tembhurne et al.^29^		Fusion of DL and ML methods with Contourlet and LBP features for skin lesion identification	Combines DL extraction with ML refinement for robustness	ISIC	0.93	Feature extraction methods must be well-defined
Waheed et al.^30^		An end-to-end system using a CNN trained on a GPU infrastructure for melanoma prediction	Utilizes GPU-based CNN for fast lesion analysis	HAM10000	0.9312	High-performance systems are required for processing
Bandy et al.^31^		Modified CNN structures with support from AI clustering to improve ROC-AUC metrics	Boosts CNN accuracy using clustering-based enhancements	ISIC 2019	0.8426	Clustering overhead may slow system response
Angeline et al.^32^		Dual-stage CNN captures ABCD-based features and uses multiple classifiers for an accuracy boost	Extracts deep features and employs multi-class classifiers	ISIC 2018	0.91	Synchronizing classifiers poses a technical hurdle
Veeramani et al.^33^		DDCNN for melanoma classification with hybrid feature fusion	Hybrid feature fusion within a double-decker CNN for improved accuracy	ISIC 2020	0.9375	High computational cost and complex architecture
Veeramani et al.^34^		YOLOv7 and XAI techniques like LIME and SHAP	Integration of YOLOv7 with XAI techniques for improved interpretability	HAM10000	0.968	Requires large annotated datasets for training.
Hu and Zhang^35^		CNNs optimized using ML-ABC and reinforcement learning to resolve data imbalance issues in melanoma classification	Uses ML-ABC strategy to balance melanoma data	ISIC 2020	0.8936	The advanced algorithm lacks detail on calibration
Kaur et al.^36^		Image refinement and DL-based segmentation followed by a classification pipeline for skin lesion identification	Integrates all stages of the CAD pipeline for melanoma	ISIC 2020	0.90184	The full CAD pipeline must be precisely managed
Adamu et al.^37^		CNN enhanced through MRFO for optimal hyperparameter tuning in skin cancer tasks	Applies MRFO to enhance parameter search in CNN	ISIC	0.8661	Processing load may increase computational demands
Dong et al.^38^		SSGNet combines colorization and classification with a shared encoder	Grid-connected multi-scale features; residual self-attention encoder	ISIC 2020	0.9129	Requires colorization during training
Zhou et al.^39^		Applying label smoothing and consistency constraints for robust pseudo-labeling in SSL	Label smoothing + consistency for noise-tolerant pseudo-labels	ISIC 2018	0.8842	Sensitive to augmentation choice
Zhu et al.^40^		Noise filtering and attentive integration for pseudo-label refinement in SSL-based skin lesion classification	NCSL for filtering + attentive clustered feature integration for feature integration	ISIC 2018	0.8715	Performance depends on the confidence threshold
Peng et al.^13^		Using MCPL and LS to dynamically refine pseudo-label thresholds, enhancing class-specific learning in SSL	Class-wise adaptive threshold + soft labels	ISIC 2018	0.85	Sensitive to pseudo-label quality
Lal et al.^41^		Self-supervised DL model augmented with DCGAN-generated dermoscopy samples	Captures features from unlabeled sets for improved modeling	ISIC	0.9786	Limited adaptability to varied imaging domains
Yuan et al.^42^		STFL filters unlabeled samples and uses focal loss to improve classification in semi-supervised skin cancer detection	Dynamic threshold + focal loss for imbalance	HAM10000	0.77	Sensitive to threshold settings
Wang^43^		Self-supervised learning, CNNs, clustering, attention	Clustering-based pseudo-labels; label-relation-aware embedding	Derm7pt	0.886	Needs high-quality paired image views
Albraika et al.^44^		DL-based architecture performing enhancement, segmentation via k-means, and capsule network-driven classification	Employs smart segmentation plus key feature isolation	ISIC	0.8975	Proper hyperparameter selection is essential
Sukanya et al.^45^		The algorithm conducts segmentation, and features are then analyzed by a DBN	Uses novel segmentation with detailed texture mapping	ISIC	0.85	Results tied to the structural setup of the algorithm
Nirmala et al.^46^		GAN-generated synthetic melanoma images with a CNN model using the VGG-16 architecture for classification	Use of GAN-generated synthetic data with CNN for improved classification and reduced preprocessing	ISIC	0.9633	Relies on synthetic data, which may not always represent real scenarios
Wang et al.^47^		Style GAN-generated images feed into a dual-branch vision transformer for melanoma detection	Enhances the variety of training data with synthetic generation	ISIC 2020	0.9792	Synthetic augmentation might introduce anomalies
Ju et al.^48^		Combination of dilated convolution layers, PPO, and GAN for robust learning on uneven datasets	Combines PPO and GAN to address imbalanced inputs	ISIC 2020	0.9843	GAN-based methods can be unstable in training
Suryanarayana et al.^49^		ResNet50-based CNN supported by a web platform offering fast diagnostic feedback	Enables fast web-based melanoma assessment	ISIC 2020	0.9948	Ongoing sample acquisition may be needed
Veeramani et al.^50^		MELIIGAN using AI-based reconstruction and stacked residual blocks	Use of hybrid loss functions and stacked residual blocks for AI-based reconstruction	ISIC 2020	0.926	Complex architecture may require high computational resources
Thanka et al.^51^		VGG16 was used for deep feature extraction; XGBoost handled the final diagnosis	Uses a hybrid pipeline for diagnostic precision	ISIC	0.934	Requires careful adjustment of pretrained networks
Shobarani et al.^52^		Multi-network deep transfer learning setup with filters and histogram techniques for preprocessing	Stacks multiple CNNs for deeper classification	ISIC 2020	0.972	Demands advanced graphics processing capabilities
Koppolu et al.^53^		EfficientNetB0 model trained on augmented dermoscopy data for lesion classification	Trains EfficientNetB0 with augmentation for skin lesion detection	HAM10000	0.9209	Augmented datasets are necessary for generalization
Orhan and Yavşan^54^		Melanoma prediction using AlexNet and MobileNet architectures prepared for mobile platform deployment	Targets mobile melanoma prediction with lightweight CNNs	8598 images	0.964	Tailored for smartphone diagnostic integration
Shakya et al.^55^		Evaluation of VGG19, ResNet18, and MobileNet models with SVM, DT, and heavy preprocessing for cancer classification	Enhances dermoscopic image quality before applying classification	ISIC 2018	0.8439	Detailed preprocessing is key to performance
Hussain et al.^56^		The Nailmelanoma-specific dataset was used to train and quantify seven CNN models for mobile applications	Deploys compressed CNNs for smartphone use	ISIC	0.9943	Application restricted to nail-focused tasks
Kalyani et al.^57^		Integration of arithmetic optimization with DL ensemble for detailed lesion analysis	Fuses metaheuristics for advanced diagnostic modeling	ISIC 2017	0.951	Complex model layers challenge configuration
Viknesh et al.^58^		Ensemble CNN and SVM with RBF kernel powering a high-accuracy classification system	Fuses dual CNNs for reliable prediction	ISIC	0.905	RBF kernel must be selected with care
Meswal et al.^59^		Weighted ensemble model combining InceptionV3, VGG16, Xception, and ResNet50 predictions	Aggregates multiple networks in a weighted combination	ISIC	0.8494	Model fusion needs an accurate performance ranking
Pérez and Ventura^60^		Active learning environment with CNN that iteratively improves via expert-guided feedback	Iteratively trains CNN with expert feedback in a loop	ISIC 2017	0.884	Relies on looped expert-involved learning
Verma et al.^61^		Detection system combining CNN-transformer hybrid with HGSO and WSA metaheuristics	Combines CNN transformer and metaheuristics for clarity and insight	HAM10000	0.89	Depends on the tuning of dedicated architectures
Vishnu et al.^62^		A pair of neural networks trained with augmented input to expand learning from limited data	Merges two networks with augmented data for learning depth	ISIC	0.91	Model learning relies on broad training input
Bazgir et al.^63^		Refined Inception network with filters to classify skin diseases, including melanoma	Applies fine-tuned Inception to distinguish skin conditions	2637 images	0.9336	Results influenced by the initial enhancement phase

### Limitations

Table 1 outlines various ML, DL, and TL techniques used for melanoma detection. Many existing studies on melanoma detection rely heavily on supervised learning techniques. While effective under ideal conditions, these methods require large, accurately labeled datasets, which are often expensive and time-consuming to obtain in the medical domain. Moreover, supervised models are susceptible to hyperparameter configurations, and their performance tends to degrade when facing class imbalance or noise in the data. TL is a promising approach to mitigating data scarcity by leveraging pre-trained models, but it also introduces new challenges. TL methods often face domain shifts when the source and target datasets differ. This problem reduces the ability of the model to generalize across different dermoscopic datasets. Also, most TL models do not handle hyperparameter tuning directly. As a result, their performance may drop due to poor parameter settings.

Several semi-supervised and self-supervised methods have been proposed to address the lack of labeled data in melanoma detection^13,38,40,42,43^. However, they often struggle with noisy pseudo-labels, poor generalization, and sensitivity to domain shifts^40^. SS-GANs provide a promising solution. They utilize adversarial training to combine labeled and unlabeled data, thereby enhancing feature learning and reducing the reliance on large annotated datasets. However, conventional SS-GANs still face limitations, including mode collapse, weak global dependency modeling, unreliable pseudo-labels, and instability under input variations. These issues reduce their robustness and limit their clinical applicability.

To overcome these issues, the proposed model integrates four targeted enhancements into the SS-GAN framework. First, a reconstruction loss is introduced into the generator to counter mode collapse and preserve structural details. Second, self-attention mechanisms are incorporated into both the generator and the discriminator. These help capture long-range dependencies and improve the quality of learned features. Third, consistency regularization is applied to the discriminator, ensuring stable predictions under augmented inputs. Fourth, confidence-based pseudo-labeling restricts supervised learning to only high-certainty predictions, improving label reliability. Additionally, an ML-ABC algorithm is used to reduce sensitivity to hyperparameter settings. It utilizes a random key representation to efficiently explore the search space. This integrated solution enhances generalization and boosts robustness on unlabeled data. It also reduces manual intervention in hyperparameter tuning, making the model highly suitable for clinical melanoma detection.

**Fig. 1:**
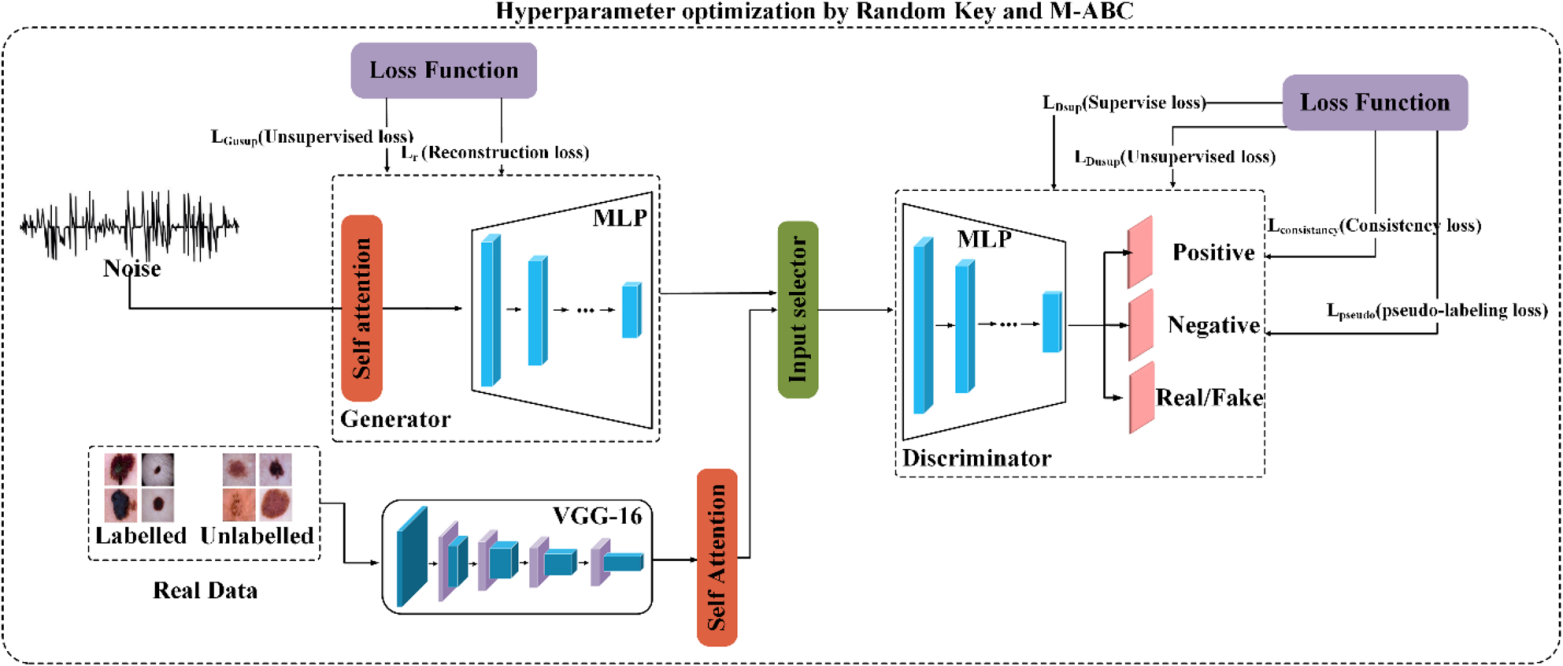
Overall architecture of the proposed melanoma detection model. The model integrates an improved SS-GAN with hyperparameter optimization based on the Random Key method and an ML-ABC algorithm.

## The proposed model

Figure 1 illustrates the overall architecture of the proposed melanoma detection model. It integrates an SS-GAN with hyperparameter optimization using the Random Key method and an ML-ABC algorithm. The model is designed as a binary classifier to distinguish between melanoma and non-melanoma cases. It utilizes both labeled and unlabeled dermoscopic images as the main input.

- Generator: A random noise vector is fed into the generator as input to produce synthetic feature vectors. This noise input is a standard feature in GAN-based models, helping to improve feature diversity and mitigate mode collapse. In our approach, the generator incorporates a novel self-attention module and a reconstruction loss ($\:{L}_{r}$). These components are not used in conventional SS-GAN architectures. The self-attention mechanism processes the input noise to model internal feature dependencies, leading to improved diversity in generated features. The reconstruction loss is designed to reduce mode collapse and enhance diversity in the generated features. Additionally, the generator employs an unsupervised loss ($\:{L}_{{G}_{usup}}$​​), which is commonly used in conventional SS-GANs. This loss guides feature generation to align with real data distributions.
- Discriminator: The discriminator receives input from two sources. The first source is synthetic feature vectors generated by the generator, which are considered fake data. The second source is feature vectors extracted from real images using the VGG-16 feature extractor to produce high-level feature embeddings. The output of the discriminator includes positive (melanoma) and negative (non-melanoma) classifications for real images, and real/fake classification for generator outputs. In our approach, the discriminator introduces novel components, including a self-attention module and additional loss functions that enhance its learning capability. The self-attention module is applied to numerical feature vectors from the generator or real image embeddings extracted by the VGG model. It captures global dependencies among features before these are passed to the multi-layer perceptron (MLP). Moreover, the incorporation of consistency loss ($\:{L}_{\text{consistency\:}}$​) ensures stable predictions on augmented inputs. The addition of pseudo-labeling loss $\:{L}_{\text{pseudo\:}}$​) leverages high-confidence predictions from unlabeled samples. Additionally, the discriminator employs the standard supervised loss ($\:{L}_{{D}_{sup}}$) for labeled data. It also uses the unsupervised loss ($\:{L}_{{D}_{usup}}$) for unlabeled data. Both losses are commonly used in conventional SS-GANs and are not part of our innovations.

### SS-GAN

GANs have significantly improved machine learning by enabling models to generate data similar to real-world examples. They are especially useful when deep learning models need to create synthetic data that resembles actual datasets. Building on this foundation, SS-GANs integrate semi-supervised learning into the GAN architecture to further enhance classification capabilities. In SS-GANs, the generator creates synthetic feature representations to challenge the discriminator. The discriminator is trained to classify samples into $\:c+1$ classes: real samples belong to one of the target classes from 1 to $\:c$, and generated (fake) samples are assigned to the extra $\:c+1$ class. Formally, let $\:G$ and $\:D$ represent the generator and discriminator, respectively. The loss function for the discriminator $\:D$ is expressed as $\:{L}_{D}\:=\:{L}_{{D}_{sup}}\:+\:{L}_{{D}_{usup}}$​​. $\:{L}_{{D}_{sup}}$​​ and $\:{L}_{{D}_{usup}}$​​ refer to the supervised and unsupervised losses, which are defined as follows:

1 $$\:{L}_{{D}_{sup}}=-{E}_{x,y\:\in\:\:{p}_{d}}\:\text{log}\left[{p}_{m}(\widehat{y}\:=\:y|x,y\:\in\:\:(\text{1,2},\dots\:,c))\right]$$

2 $$\:{L}_{{D}_{usup}}=-{E}_{x\:\in\:\:{p}_{d}}\:log\left[1-{p}_{m}\left(\widehat{y}\:=\:y|x,y\:=\:c\:+\:1\right)\right]{-E}_{x\:\in\:\:G}\:\text{l}\text{o}\text{g}\left[{p}_{m}\right(\widehat{y}\:=\:y|x,y=c+1)]$$

Here, $\:x$ denotes an input sample, and $\:y$ is the true label associated with $\:x$ in labeled data. $\:{p}_{G}$ and $\:{p}_{D}$​ denote the distributions of generated and real data, respectively. The symbol $\:E$ denotes the expected value, while $\:log$ refers to the natural logarithm. Furthermore,$\:\widehat{y}\:$​ represents the predicted label for sample $\:x$. $\:{p}_{m}(\widehat{y}\:=\:y|x,y\:=\:c\:+\:1)$ indicates the probability that sample $\:x$ belongs to the fake class. In contrast, $\:{p}_{m}(\widehat{y}\:=\:y|x,y\:\in\:\:(\text{1,2},\dots\:,c))$ represents the probability that $\:x$ belongs to one of the real target classes.

The generator loss function minimizes the probability that the discriminator classifies synthetic samples as part of the additional fake class ($\:c\:+\:1$):

3 $$\:{L}_{{G}_{usup}}={-E}_{x\:\in\:\:G}\:log[1-{p}_{m}\left(\widehat{y}\:=\:y|x,y\:=\:c\:+\:1\right)]$$

#### Structure of the generator and discriminator

In the proposed SS-GAN architecture, both the generator and discriminator employ MLP networks to model features and perform classification. Each network receives an input feature vector of dimension $\:d$. Specifically, the generator takes a noise vector sampled from a normal distribution and transforms it into a feature vector $\:{F}_{fake}\in\:{R}^{d}$. The discriminator accepts an input vector $\:{F}^{\text{*}}\in\:{R}^{d}$, which can be either $\:{F}_{fake}$​, generated by the generator, or a real feature vector $\:{F}_{VGG}$​ extracted from real images using the VGG16 model. In our implementation of VGG16, the receptive field of the final convolutional layer is approximately 212 × 212 pixels, which nearly covers the entire input image of 224 × 224 pixels. This ensures that each extracted feature vector encompasses a broad spatial context, which is critical for accurate downstream classification by the discriminator. The receptive field size at layer l can be computed recursively using the following formula:

4 $$\:{R}_{l}={R}_{l-1}+\left({k}_{l}-1\right)\times\:\prod\:_{i=1}^{l-1}\:{s}_{i}$$

In this formula, $\:{R}_{l}$ is the receptive field of the previous layer, $\:{k}_{l}$ is the kernel size at layer $\:l$, and $\:{s}_{i}$ denotes the stride of the $\:i$-th layer from 1 to $\:l-1$. For the VGG16 architecture, this yields a receptive field of 212 × 212 at the final convolutional layer.

##### Self-attention layer

A self-attention mechanism is added before the MLP layers in both the generator and discriminator. This mechanism helps the model capture dependencies between distant components of the input features. In the generator, it promotes better coordination among different parts of the generated feature vectors, improving their diversity and consistency. In the discriminator, this mechanism improves feature representation by focusing on the most relevant global feature interactions.

The mechanism takes the input matrix $\:X\in\:{\mathbb{R}}^{n\times\:d}$, where $\:n$ is the number of elements, and produces query ($\:Q$), key ($\:K$), and value ($\:V$) matrices using learned weight matrices. The attention operation calculates weighted combinations of these matrices while keeping the feature dimension d unchanged. This allows both networks to capture the global context. Maintaining the feature dimension helps the attention output capture long-range relationships before the MLP layers perform their nonlinear transformations.

Mathematically, the query, key, and value matrices are computed as follows:

5 $$\:Q=X{\times\:\text{W}}_{Q},K=X\times\:{\text{W}}_{K},V=X{\times\:\text{W}}_{V\:}$$

where $\:{W}_{Q},{W}_{K},{W}_{V}\in\:{\mathbb{R}}^{d\times\:{d}_{k}}$​. The attention output is then calculated as:

6 $$\:\text{A}\text{t}\text{t}\text{e}\text{n}\text{t}\text{i}\text{o}\text{n}(Q,K,V)=\text{S}\text{o}\text{f}\text{t}\text{m}\text{a}\text{x}\left(\frac{Q{\times\:K}^{T}}{\sqrt{{d}_{k}}}\right)\times\:V$$

Here, $\:{d}_{k}$​, the dimensionality of the key vectors, serves as a scaling factor to prevent the dot products from becoming excessively large. The softmax function converts values into a probability distribution, assigning higher weights to more relevant key-query pairs. It is mathematically defined as:

7 $$\:\text{S}\text{o}\text{f}\text{t}\text{m}\text{a}\text{x}\left({z}_{i}\right)=\frac{{e}^{{z}_{i}}}{\sum\:_{j=1}{e}^{{z}_{j}}}$$

where $\:{z}_{i}$ is the $\:i$-th element of the input vector $\:z$. This operation transforms the input values into probabilities, ensuring that each output value lies between 0 and 1 and that the sum of all output values equals 1. These normalized values can then be used as attention weights.

#### Loss function

##### Generator

Traditional generators in SS-GANs often suffer from mode collapse, a problem where the generator produces limited and repetitive samples. This reduces the diversity of generated data and limits the ability of the model to capture the full complexity of real data. As a result, mode collapse causes a poor generalization and weaker classification performance in tasks such as melanoma detection.

To tackle this issue and improve training stability, our enhanced SS-GAN incorporates a reconstruction loss into the objective function of the generator. This is done in conjunction with the conventional adversarial loss. The reconstruction loss encourages the generator to produce outputs that closely resemble real samples in the feature space, thereby preserving structural details and encouraging diversity. Formally, the reconstruction loss is defined as:

8 $$\:{L}_{r}={E}_{x\:\in\:\:{p}_{x}}\:{{\parallel}G\left({D}_{F}\right(x\left)\right)\:-\:x\parallel}_{2}$$

In this configuration, x represents a real input sample sampled from the real distribution $\:\:{p}_{x}$​. The symbol $\:{.\parallel\parallel}_{2}$ denotes the L2 norm. $\:{D}_{F}$​ refers to the discriminator without its final layer, which is used to convert the input into a high-level feature space. The term $\:G\left({D}_{F}\right(x\left)\right)\:$means that $\:{D}_{F}$​ extracts feature representations from the input, and $\:G$ reconstructs these features into data resembling the original input. Ultimately, the loss function of the generator is established as follows:

9 $$\:{L}_{G}={L}_{{G}_{usup}}+\lambda\:\:\times\:\:{L}_{r}\:$$

where $\:\lambda\:$ represents a parameter that adjusts the influence of the reconstruction loss. The reconstruction loss ($\:{L}_{r})$ is crucial for stabilizing SS-GAN training. It helps directly prevent mode collapse. This loss measures the difference between original data samples and their reconstructions. The generator creates these reconstructions after encoding data through the feature-extraction layer of the discriminator ($\:{D}_{F}$). Minimizing this difference ensures the generator produces samples that closely resemble real data and preserves their structure and function. This approach helps prevent mode collapse by encouraging the generator to produce diverse outputs instead of repeating limited patterns. The inclusion of reconstruction loss in the objective of the generator fosters a more balanced training process. The parameter $\:\lambda\:$ controls the balance between reconstruction accuracy and other training goals, ensuring that generated samples remain authentic and diverse during training. Applying reconstruction loss in this manner addresses common GAN training instability and significantly enhances model robustness.

##### Discriminator

Traditional discriminators in SS-GAN frameworks face several challenges that can reduce their accuracy and stability. A major problem is their sensitivity to small changes in input, which causes unstable predictions. This instability prevents the discriminator from reliably distinguishing between real samples and generated ones, which in turn lowers the overall model performance. Also, when working with unlabeled data, discriminators can assign wrong pseudo-labels. This leads to error propagation and weakens training. To address these problems, two key techniques are integrated into the discriminator:

- Consistency regularization: This technique enforces the discriminator to produce stable predictions even when inputs are perturbed or augmented. It works by reducing the difference between the outputs of the discriminator on the original and augmented versions of a sample. Formally, the consistency loss is defined as:

10 $$\:{L_{{\text{consistency}}\:}} = {\mathbb{E}_{x\:\approx{p_d}}}\parallel {p_m}\left( {\hat y|x} \right) - {p_m}\left( {\hat y|\mathop x\limits^\sim } \right){\parallel ^2}$$

Here, $\:x$ represents the original sample, $\:\stackrel{\sim}{x}$ denotes the augmented version of that sample, and $\:{p}_{m}$​ refers to the probability distribution predicted by the discriminator.

- Confidence-based pseudo-labeling: For unlabeled data, only samples with high-confidence predictions—those whose highest predicted class probability is above a threshold $\:\tau\:$—are used in supervised training. This selective training helps reduce the impact of incorrect pseudo-labels and improves learning reliability. The pseudo-label loss is given by:

11 $$\:{L}_{\text{pseudo\:}}=-{\mathbb{E}}_{x\sim\:{p}_{\text{unlabeled\:}}}1\left[\underset{y}{max}\:{p}_{m}(\widehat{y}=y|x)\ge\:\tau\:\right]\times\:\text{l}\text{o}\text{g}{p}_{m}\left(\widehat{y}={\widehat{y}}_{\text{pseudo\:}}|x\right)$$

Here, $\:1(\cdot\:)$ is the indicator function that selects only confident samples, meaning it outputs 1 when the condition inside is true and 0 otherwise. The variable $\:x$ denotes an unlabeled data sample. This sample is drawn from the distribution of unlabeled data, represented by $\:{p}_{\text{unlabeled\:}}$​. The term $\:\mathop {max}\limits_y \:{p_m}\left( {\hat y = y|x} \right)$ finds the highest predicted probability across all classes $\:y$ for the sample $\:x$. This prediction is given by the probability distribution of the discriminator $\:{p}_{m}$​. The threshold $\:\tau\:$ is a predefined confidence level used to determine whether a sample is sufficiently confident to be included in supervised training. The term $\:{\widehat{y}}_{\text{pseudo\:}}$ is the pseudo-label assigned to $\:x$, corresponding to the class $\:y$ with the highest predicted probability. The overall discriminator loss combines supervised and unsupervised components, along with the consistency and pseudo-label losses:

12 $$\:{L}_{D}^{\text{final\:}}={L}_{{D}_{\text{sup\:}}}+{L}_{{D}_{\text{usup\:}}}+{\lambda\:}_{\text{consistency\:}}\times\:{L}_{\text{consistency\:}}+{\lambda\:}_{\text{pseudo\:}}\times\:{L}_{\text{pseudo\:}}$$

Here, $\:{\lambda\:}_{\text{consistency\:}}$ and $\:{\lambda\:}_{\text{pseudo\:}}$control the relative importance of the consistency and pseudo-label losses, respectively.

##### Configuration

During the back-propagation phase, only unlabeled samples that are misclassified into the additional class $\:c+1$ contribute to the unsupervised loss $\:{L}_{{D}_{usup}}$​​. In other cases, their impact on the loss is excluded to neutralize their influence. In contrast, labeled samples directly contribute to the supervised loss $\:{L}_{{D}_{sup}}$​​. Generated samples from the generator influence both $\:{L}_{D}$ and $\:{L}_{G}$​. The discriminator is penalized if it misclassifies these samples as real. Likewise, the generator (G) is penalized if it fails to fool the discriminator. During the training of the discriminator, the weights in the VGG-16 backbone are updated. This improves the feature representation of the model by using both labeled and unlabeled data.

The consistency regularization used in the discriminator involves creating an augmented version$\:\:\stackrel{\sim}{x}$ of each sample $\:x$. Common transformations include random flipping, rotation, or adding Gaussian noise. The discriminator is then encouraged to produce consistent predictions for both $\:x$ and $\:\stackrel{\sim}{x}$. This improves robustness to small changes in input. In the pseudo-labeling strategy, only high-confidence unlabeled samples are added to the supervised training. These are samples where the predicted class probability exceeds a predefined threshold $\:\tau\:$. Such samples are treated as labeled and contribute to $\:{L}_{\text{pseudo\:}}$​. This selective process reduces the chance of learning from noisy pseudo-labels and ensures that synthetic supervision is reliable.

Once training is complete, the generator is discarded, and the discriminator is retained for classification. The model preserves the original functionality of the VGG-16 architecture for inference. This strategy ensures that inference has the same computational cost as the standard VGG-16 model. It avoids any increase in computational overhead.

**Table 2:** Optimized generator and discriminator hyperparameters with search ranges and best values.

Hyperparameter	Range	Best value	
		Generator	Discriminator
Batch size	[16, 512]	ISIC-2020 (62), and HAM10000 (55)	ISIC-2020 (68), and HAM10000 (60)
Epoch	[64, 1024]	ISIC-2020 (252), and HAM10000 (213)	ISIC-2020 (242), and HAM10000 (198)
Learning rate	[0–1]	ISIC-2020 (0.0001), and HAM10000 (0.0002)	ISIC-2020 (0.0004), and HAM10000 (0.0005)
Activation function	[ReLU, Leaky ReLU, Linear, Tanh, Sigmoid]	ISIC-2020 (ReLU), and HAM10000 (ReLU)	ISIC-2020 (ReLU), and HAM10000 (ReLU)
Dropout rate	[0–1]	ISIC-2020 (0.62), and HAM10000 (0.52)	ISIC-2020 (0.68), and HAM10000 (0.55)
Number of layers in MLP	[1, 10]	ISIC-2020 (5), and HAM10000 (3)	ISIC-2020 (4), and HAM10000 (3)
Noise size	[16, 1024]	ISIC-2020 (214), and HAM10000 (189)	NA
$\:\lambda\:$	[0–1]	ISIC-2020 (0.42), and HAM10000 (0.38)	NA
$\:{\lambda\:}_{\text{consistency\:}}$	[0–1]	NA	ISIC-2020 (0.28), and HAM10000 (0.39)
$\:{\lambda\:}_{\text{pseudo\:}}$	[0–1]	NA	ISIC-2020 (0.41), and HAM10000 (0.35)

**Fig. 2:**
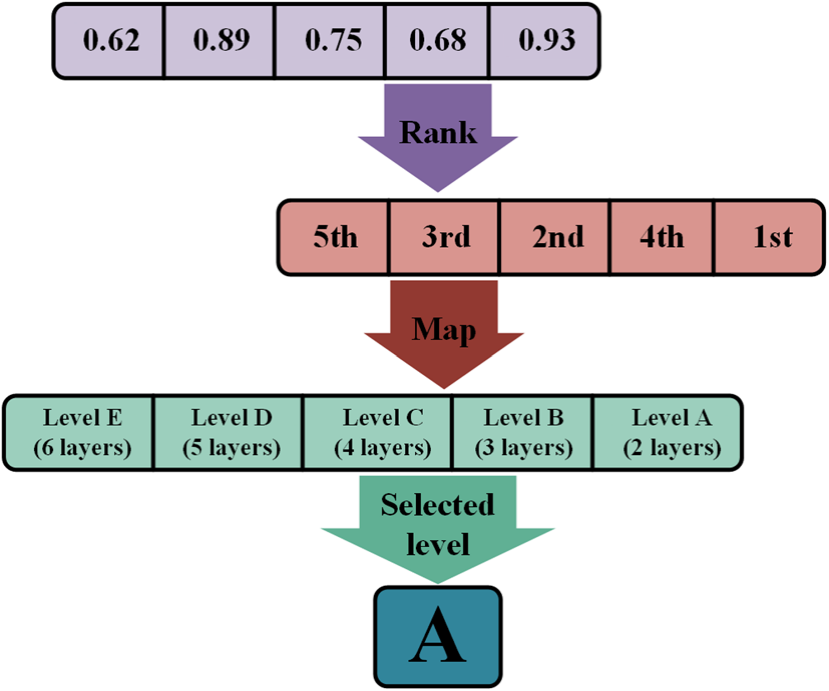
Random Key encoding example showing ranking, mapping, and final level selection.

### Hyperparameter optimization

Optimizing hyperparameters is crucial for SS-GAN models, as it significantly impacts their training stability and output quality. Proper tuning helps the model converge faster. It also reduces training time and prevents common issues, such as mode collapse. By selecting optimal values for the learning rate, batch size, and architecture-specific parameters, the SS-GAN can generate more realistic and diverse outputs. Hyperparameter optimization helps the model learn the underlying data distribution effectively. This is essential for applications that need high-fidelity generative results, such as melanoma detection.

Table 2 displays the hyperparameters that were adjusted during our study. We established a suitable range of values for each hyperparameter. These ranges were based on recommendations from deep-learning research related to melanoma.

#### Random key

This paper uses the Random Key approach for hyperparameter optimization in the proposed SS-GAN. This approach simplifies the encoding of parameter settings into a continuous domain. As a result, it becomes easier to apply metaheuristic optimization algorithms. This technique improves the robustness and efficiency of the tuning process. It also enables a broader exploration of the hyperparameter space and enhances convergence. It effectively aligns with the requirements of SS-GAN for adapting to diverse data characteristics. It helps achieve a finely tuned model with superior performance in semi-supervised learning scenarios.

The process of the Random Key method is broken down into the following steps:

- Population initialization: A population of $\:T$ real-valued vectors is generated. Each vector has$\:\:D$ dimensions. These vectors are denoted as $\:{p}_{1},\:{p}_{2},\:...,\:{p}_{T}$. Each vector represents a candidate solution or a possible hyperparameter configuration.
- Hyperparameter mapping design: Suppose the model has $\:C$ hyperparameters. Each hyperparameter, denoted as c and ranging from 1 to $\:C$, is assigned a sub-vector of length $\:{D}_{c}$. If the parameter is continuous, $\:{D}_{c}$ is set to 1. For categorical parameters, $\:{D}_{c}$ can be greater than 1 to support sorting-based encoding. The total dimensionality of each vector is denoted as $\:D=\sum\:_{c=1}^{C}{D}_{c}$.
- Encoding and decoding mechanism: Each individual$\:\:{p}_{i}$ is partitioned into $\:C$ segments. For categorical hyperparameters, each segment is sorted in descending order. The index of the highest value is then used to select an option from a predefined category list, denoted as $\:{MAP}_{c}$. For continuous hyperparameters, the real value in the corresponding position is used directly.
- Application of evolutionary operators: The ABC algorithm updates the positions of real-valued vectors using strategies inspired by bee foraging behavior. These strategies include neighbor-based exploration, probabilistic selection of better solutions, and random search mechanisms to ensure diversity. The Random Key scheme ensures that after these updates, the decoded configurations remain valid and meaningful.

An example illustration of the Random Key encoding process is shown in Fig. 2. This example considers a categorical hyperparameter that represents the number of layers in the model. It can take five discrete values. Each value is linked to a specific level label. Level A represents two layers. Level B corresponds to three layers. Level C indicates four layers. Level D maps to 5 layers. Level E represents six layers. Consider the real-valued sub-vector of size $\:{D}_{c}=5$ as [0.62, 0.89, 0.75, 0.68, 0.93]. In the ranking step, elements of the vector are sorted in ascending order. The ranked vector is then passed to the mapping step to select the appropriate level. The position of the top-ranked value, 0.93 in this case, determines the selected category. This value corresponds to the first position in the mapped array. As a result, the selected level is level A, which represents a configuration with two layers.

#### ABC

We utilize the ABC algorithm to optimize the Random Key method due to its strong capabilities for both exploration and exploitation. These capabilities are essential for navigating complex and high-dimensional hyperparameter spaces. This approach enables a more systematic and thorough search. It reduces the risk of premature convergence and improves the overall parameter-tuning process. The ABC algorithm can adapt its search strategy based on the fitness landscape. This adaptability makes it especially effective for optimizing the Random Key method in SS-GAN training.

The ABC algorithm revolves around two main concepts: food sources and artificial bees. Artificial bees simulate the natural foraging behavior of real bees. Each food source represents a potential solution to an optimization problem. Bees are divided into employed, onlooker, and scout bees. Employed bees explore known sources to find richer nectar and share results with onlookers through dances in a designated area. The colony maintains an equal split between employed and onlooker bees. Each employed bee is linked to one hive. Onlooker bees use shared insights to explore adjacent areas. If employed bees fail repeatedly, they become scout bees to search for new regions^64^.

The ABC algorithm operates through four iterative phases: initialization, employed bee phase, onlooker bee phase, and scout bee phase. Each phase ends when a specific termination condition is met. This cyclic process ensures steady advancement towards the optimal solution. In the initialization phase, the food sources are placed based on the bee positions. These positions are defined within a designated search area, as explained below^65^:

13 $$\:{x}_{i}^{j}={x}_{min}^{j}+rand\left(\text{0,1}\right)({x}_{max}^{j}-{x}_{min}^{j})$$

where $\:{x}_{i}^{j}\:$shows the initial value of dimension $\:i$ in candidate solution $\:j$. Values $\:{x}_{min}^{j}$ and $\:{x}_{max}^{j}$ denote the minimum and maximum bounds for dimension $\:i$ in solution $\:j$, respectively. $\:rand\left(\text{0,1}\right)$ is a random number uniformly distributed between 0 and 1. It ensures that $\:{x}_{i}^{j}$ lies within the interval $\:[{x}_{min}^{j},\:{x}_{max}^{j}]$.

In the employed bee phase, bees evaluate the current food sources and identify more promising ones. These better options replace the underperforming sources, as described below^65^:

14 $$\:{v}_{i}^{j}={x}_{i}^{j}+{\phi\:}_{i}^{j}({x}_{i}^{j}-{x}_{k}^{j})$$

In this equation, $\:k$ represents an index randomly selected from the colony. The term $\:{\phi\:}_{i}^{j}$ represents a random coefficient that ranges between − 1 and 1. The variable $\:{v}_{i}$ refers to the newly adjusted potential food source, which is derived by modifying the original $\:{x}_{i}$.

During the onlooker bee phase, employed bees share their discoveries with onlooker bees. The onlooker bees then use a probabilistic method to select and evaluate solutions based on quality. This process helps update the solution pool. The probability of an onlooker bee opting for a new solution is given as follows^65^:

15 $$\:{p}_{i}=\frac{fit\left({x}^{j}\right)}{{\sum\:}_{n=1}^{BN}fit\left({x}^{n}\right)}$$

where $\:fit\left({x}^{j}\right)$ denotes the fitness of the $\:{j}^{th}$ solution, and $\:BN$ is the overall number of solutions. Scout bees identify solutions that do not improve after several iterations. They replace these underperforming solutions with new exploratory candidates.

##### Mutual learning-based ABC

In the standard ABC algorithm, bees choose the position of a food source at random. They then modify this position to generate a new one. If this new position yields a better fitness value, it replaces the existing position. If not, the original position is retained. In multi-dimensional optimization tasks, one dimension is selected randomly, and its value is modified. The solution with better performance is then adopted in that iteration. According to Eq. 14, the generation of a new solution depends only on two parameters, $\:\varphi\:$ and $\:\theta\:$. This limited dependency makes the quality of new food sources unpredictable. Some new solutions improve the existing one, while others degrade it. The ABC algorithm strives to uncover food sources with superior fitness values.

To improve this process, an enhanced version of ABC is proposed. It incorporates a mutual learning mechanism that enables bidirectional knowledge exchange between current and neighboring food sources. The standard ABC generates new solutions through stochastic perturbations alone. In contrast, the enhanced method uses fitness comparisons to guide the solution updates. Specifically, if a neighboring food source has a better fitness value, the current candidate learns from it and moves in its direction. Conversely, if the current candidate is better, it retains its strength while still exploring minor variations through interaction. This strategy improves exploitative accuracy by focusing the search around promising areas. It also preserves the exploratory nature of ABC through stochastic variation. This informed decision-making reduces the randomness of updates. It promotes faster convergence and improves the quality of evolved solutions. As a result, the enhanced ABC algorithm not only boosts model stability but also contributes directly to more effective feature extraction, leading to improved classification performance in the semi-supervised learning setting.

The proposed mutual learning approach is defined as follows^65^:

16 $$\:{\:\:\:v}_{i}^{j}=\left\{\begin{array}{c}{x}_{i}^{j}+{\phi\:}_{i}^{j}\left({x}_{k}^{j}-{x}_{i}^{j}\right),\:\:\:{Fit}_{i}<{Fit}_{k}\\\:{x}_{k}^{j}+{\phi\:}_{i}^{j}\left({x}_{i}^{j}-{x}_{k}^{j}\right),\:\:\:{Fit}_{i}\ge\:{Fit}_{k}\end{array}\right.$$

Here, $\:{Fit}_{i}$ and $\:{Fit}_{k}\:$denote the fitness levels of neighboring and current food sources, respectively. The variable $\:{\phi\:}_{i}^{j}$ is a random coefficient selected from the range between 0 and $\:F$. The value $\:F$ denotes the mutual learning coefficient and is always positive. This method enhances solutions by favoring food sources with higher fitness. The value of $\:F$ is pivotal in improving and maintaining the quality of solutions. As the value of $\:F$ increases, it reduces variability in the solution updates. This reduction indicates movement toward the enhanced fitness of a neighboring food source. However, when $\:F$ becomes excessively large, it can disrupt the essential balance between exploration and exploitation. This disruption may compromise the overall efficiency of the optimization process.

Traditional cooperative co-evolutionary algorithms split problems into smaller components and evolve them separately. The enhanced ABC model introduces a mutual learning strategy for interactive, integrated learning. In standard co-evolution, individuals evolve based on local objectives with little interaction, contributing to a global solution in isolation. Mutual learning enables each candidate solution to adjust its trajectory dynamically by utilizing information shared by better-performing neighbors. This real-time exchange improves collective adaptation, reduces redundancy, and prevents stagnation. Compared to Bayesian optimization (BO) or evolutionary alternatives like covariance matrix adaptation evolution strategy (CMA-ES)^66^ML-ABC achieves faster convergence and stronger exploration of high-dimensional spaces through effective knowledge sharing.

Furthermore, the mutual learning mechanism in the enhanced ABC algorithm plays an essential role in preventing entrapment in local optima. This is achieved through a structured exchange of information among candidate solutions. Each updates its position based on the fitness difference with neighboring candidates rather than using arbitrary movements. Weaker candidates are guided adaptively toward stronger ones. This process allows the population to gradually move toward more optimal regions of the solution space. This adjustment encourages directional learning instead of blind exploration. It accelerates convergence and enhances diversity by allowing several high-potential regions to emerge and evolve in parallel. As a result, the population avoids premature convergence around suboptimal peaks. It also preserves a balance between global exploration and local exploitation, both of which are essential for effective optimization in complex and high-dimensional problem spaces.

**Fig. 3:**
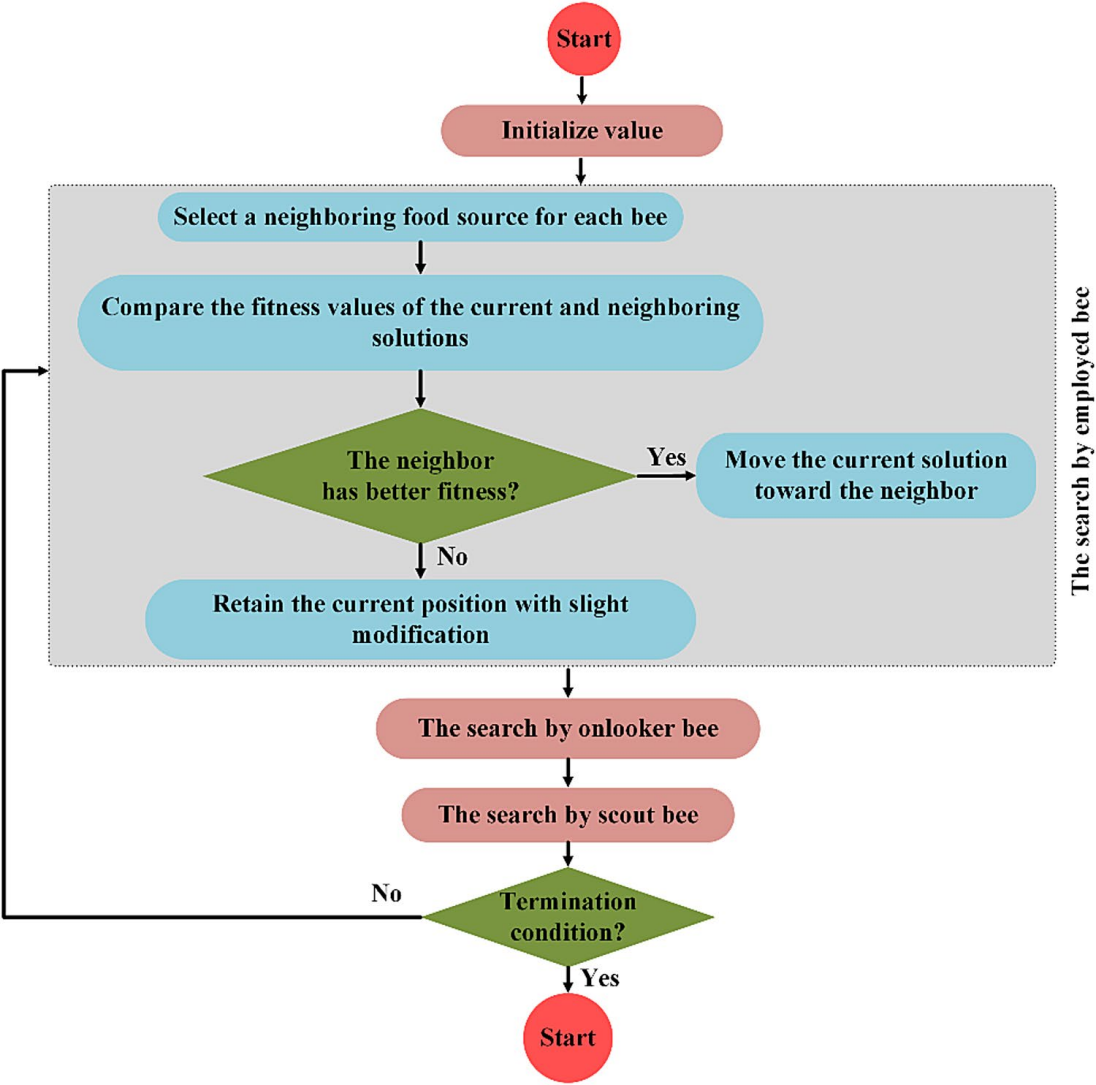
Overview of the mutual learning-based ABC algorithm phases.

Figure 3 provides an extensive depiction of the mutual learning-based ABC algorithm. First, the algorithm generates a population of bees with random positions within the defined search space. Then, in the employed bee phase, each bee selects a neighboring solution and evaluates its fitness. If the neighboring solution has better fitness, the bee updates its position by moving toward it. The fitness difference determines the step size and direction during the update. If the neighbor is less fit, the bee retains its current position while applying a slight variation to encourage local exploration. The mutual learning mechanism promotes directional movement through informed comparisons. This helps balance exploitation and exploration while improving convergence. The rest of the algorithm remains consistent with the original ABC framework. During the onlooker bee phase, bees select food sources based on probability related to fitness. They then apply the same update rules used in the employed bee phase. In the scout bee phase, stagnant solutions are replaced with the same update rules used in the employed bee phase. The cycle repeats until the termination condition is satisfied.

**Fig. a:**
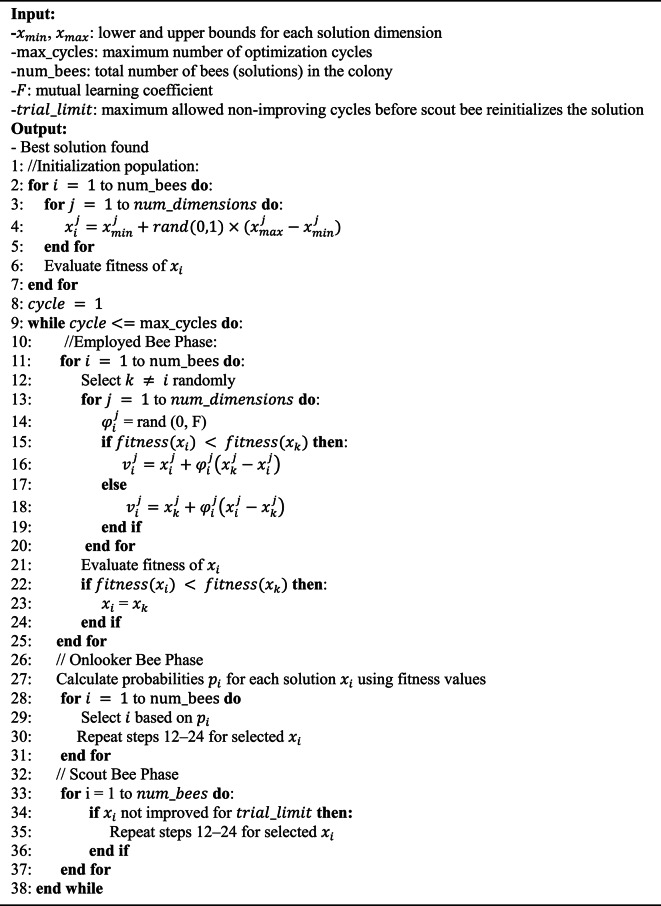
Overview of the mutual learning-based ABC algorithm phases.

###### Algorithm 1

presents the step-by-step pseudocode for the proposed ML-ABC algorithm. The optimization process begins with the initialization phase, where a population of candidate solutions, referred to as bees, is randomly generated. Each solution vector is constructed by assigning values drawn uniformly from the specified range in every dimension. This range is defined by the lower and upper bounds, denoted as $\:{x}_{min}\:$and $\:{x}_{max}$. The parameter $\:\text{n}\text{u}\text{m}\_\text{b}\text{e}\text{e}\text{s}$ defines the number of candidate solutions. The algorithm evolves these solutions iteratively to find the one with the highest fitness.

After initialization, the main optimization loop runs for a maximum number of iterations defined by $\:\text{m}\text{a}\text{x}\_\text{c}\text{y}\text{c}\text{l}\text{e}\text{s}$. In each cycle, the algorithm proceeds through three main stages. These include the employed bee phase, the onlooker bee phase, and the scout bee phase. In the employed bee phase, each bee selects a neighboring solution. This neighbor is indexed as $\:k$, where $\:k\:\ne\:\:i$. The bee compares the fitness values of its current solution and the neighbor. A random coefficient $\:{\phi\:}_{i}^{j}$ is selected from the interval $\:[0,\:F]$. Here, $\:F$ is a user-defined mutual learning coefficient that controls the update scale. If the neighbor has higher fitness, the bee moves toward it using a directional update. If the fitness of the neighbor is not higher, the update is reversed. This allows the bee to retain its current position while introducing slight exploratory variation. This strategy promotes informed search behavior and balances the exploitation of good solutions with localized exploration.

Next, in the onlooker bee phase, the algorithm assigns selection probabilities to each solution based on its fitness value. These probabilities determine which bees are selected for further updates. The same mutual learning rule used in the employed bee phase is applied again. This probabilistic mechanism focuses the efforts of the algorithm on promising areas of the search space. At the same time, it maintains diversity across the population of solutions.

In summary, the ML-ABC algorithm systematically updates its population through repeated cycles of learning, selection, and reinitialization. The algorithm uses mutual learning, which is guided by the fitness differential and scaled by the $\:F\:$coefficient. This approach improves convergence speed while preserving the ability to explore the global search space.

**Table 3:** Summary of ISIC-2020 and HAM10000 datasets utilized in this research.

Dataset Feature	ISIC-2020	HAM10000	PH2	DermNet
Data origin	ISIC archiveSourced from the ISIC archive	Compiled by the Medical University of Vienna and Skin Cancer Clinic in Queensland	Pedro Hispano Hospital, Portugal	Open-access dermatology repository
Total number of images	44108 samples	10015 samples	200 samples	Over 23,000 images
Types of skin conditions	Includes melanoma along with various other lesion types	Encompasses seven different categories of skin disorders	Two categories: melanoma and non-melanoma	23 dermatological conditions including melanoma
Training data size	30875 samples (approx. 70%)	7010 samples (approx. 70%)	Not used for training	Not used for training
Validation data size	6616 samples (approx. 15%)	1502 samples (approx. 15%)	Not used for validation	Not used for validation
Test data size	6617 samples (approx. 15%)	1503 samples (approx. 15%)	All 200 samples used for test	All samples used for test
Melanoma-specific instances	1854 in training set, 397 in validation set, 397 in test set	779 in training set, 167 in validation set, 167 in test set	40	2300
Non-melanoma instances	29021 in training set, 6219 in validation set, 6220 in test set	6231 in training set, 1335 in validation set, 1336 in test set	160	20700
Labeled data	21612 samples (approx. 70% of training data)	4907 samples (approx. 70% of training data)	Not used for training	Not used for training
Unlabeled data	9263 samples (approx. 30% training data)	2103 samples (approx. 30% training data)	Not used for training	Not used for training
Population representation	Wide-ranging global dataset with variety in ethnicity and gender	Balanced gender, broad range of ages and body regions	Single-institution dataset; uniform imaging	Broad real-world diversity across skin tones
Additional information	One of the most extensive dermoscopy datasets; global scope supports generalization	Collected over time under varied imaging conditions for broader analysis	Standardized acquisition; used for generalization testing	Public resource; used for generalization testing

## Experimental results

This section starts with an in-depth dataset analysis, examining its characteristics and their relevance to our study. After the dataset examination, the section describes the evaluation metrics in detail. These metrics define the primary benchmarks and criteria used to assess the performance of the model. The section concludes with the presentation of experimental results. It highlights key findings and discusses their implications for the study objectives.

### Datasets

This study utilized two prominent datasets to evaluate the performance of the proposed model. The first dataset originates from the SIIM-ISIC Melanoma Classification Challenge. It is included in the publicly accessible ISIC-2020 dataset, which is hosted on Kaggle. This dataset is part of the International Skin Imaging Collaboration (ISIC) Archive^67^. The archive is known as the largest open-source collection of high-resolution dermoscopic images of skin lesions. The dataset includes samples from regions such as North America, Europe, and Asia. This international coverage supports the applicability of the model across diverse population groups. It comprises images collected from nearly 2,056 individuals, with 33,126 samples designated for training and 10,982 for testing. It covers a wide range of ethnic and gender groups. This diversity ensures balanced evaluation and reduces bias toward any specific population segment.

The second dataset examined in this research is HAM10000 ^68^. It is recognized as one of the largest resources currently available for dermoscopic image analysis. This dataset was collected using images captured by multiple imaging devices under varied environmental conditions. This dataset was chosen because it includes many clinically relevant skin lesion types. It provides a wide variety of lesion categories that reflect real-world clinical diversity. It has also been widely adopted as a benchmark in melanoma detection studies. Its widespread use enables fair comparison with state-of-the-art models and contributes to the reproducibility and clinical validation of results. HAM10000 was compiled over 20 years through collaboration between the Department of Dermatology at the Medical University of Vienna and the Skin Cancer Clinic in Queensland, Australia. HAM10000 contains 10,015 dermoscopic images representing seven diagnostic categories. These include 6,705 samples of melanocytic nevi (nv), 1,113 of melanoma (mel), 1,099 of benign keratosis (bkl), 514 of basal cell carcinoma (bcc), 327 of actinic keratoses (akiec), 142 of vascular anomalies (vasc), and 115 of dermatofibroma (df). The dataset includes samples from both male and female individuals. It consists of 5,406 male samples and 4,552 female samples, covering different anatomical sites and age groups.

To evaluate the generalization capabilities of the proposed model, we conducted experiments using the PH2 ^69^ and DermNet^70^ datasets. The PH2 dataset was developed by the Dermatology Service of Pedro Hispano Hospital in Matosinhos, Portugal. It is a valuable resource for researchers and clinicians aiming to improve diagnostic precision in dermatology. This dataset al.so serves as a standardized platform for developing and validating diagnostic tools. All PH2 images were captured using the Tuebinger Mole Analyzer system. This system includes a 20× magnification lens that ensures consistency across images. The images are stored as BMP files in RGB color format, each with a resolution of 768 × 560 pixels. The dataset contains 200 images, categorized into 40 melanoma cases and 160 non-melanoma cases. DermNet is a large public dataset that contains over 23,000 expert-verified images. These images cover 23 distinct dermatological conditions, such as melanoma, eczema, psoriasis, acne, and vascular tumors.

Although four datasets are widely adopted benchmarks, it is important to acknowledge their limitations. ISIC-2020 and HAM10000 do not include explicit metadata on skin phototypes based on the Fitzpatrick scale. This limitation restricts comprehensive performance assessment across skin tones, particularly underrepresented darker types (IV-VI). The PH2 dataset follows strict imaging standards. In contrast, ISIC-2020, HAM10000, and DermNet include images taken under heterogeneous and less controlled conditions. These conditions result in variations in lighting, resolution, and imaging equipment. DermNet differs from ISIC and HAM10000 by contributing more diversity. It includes a wider range of dermatological conditions across varied skin tones and real-world photographic environments. This diversity provides useful, though indirect, insights into the behavior of the model on broader populations. Despite this improvement, the absence of standardized phototype annotation across all datasets remains a significant limitation. Future research should focus on external validation using datasets with clearly labeled skin types. These datasets should also use standardized acquisition settings to support stronger claims of generalizability.

Table 3 presents an overview of the four datasets applied in this research. To ensure a uniform classification framework across all datasets, we focus on a binary classification task (melanoma vs. non-melanoma). For datasets with multiple lesion categories, such as HAM10000, all non-melanoma lesion types are merged into a single “non-melanoma” class. This grouping ensures consistent evaluation across datasets with different labeling schemes. It also reflects a practical clinical scenario where the primary goal is to differentiate melanoma from other benign or less critical skin conditions. This approach simplifies the modeling process. It also enables the proposed SS-GAN framework to learn generalized features that are effective for melanoma versus non-melanoma tasks across diverse datasets.

The ISIC-2020 and HAM10000 datasets were fully used in this study for training, validation, and testing purposes. 70% of the samples from each class were used for training. The remaining 30% was divided equally, with 15% for validation and 15% for testing. The 70-15-15 split was selected to balance effective model training with sufficient data for tuning and testing. Using 70% of the data for training exposes the model to a wide variety of examples. This improves learning and enhances generalization capabilities. The 15% validation set helps fine-tune model parameters and reduces the risk of overfitting. The final 15% used for testing provides an unbiased method for evaluating model performance in real-world settings.

In the semi-supervised stage, we used a hybrid strategy. About 30% of the training data was labeled, while the remaining 70% was unlabeled. This approach is widely used in semi-supervised learning, as it balances the use of scarce labeled data with the abundance of unlabeled data. The labeled data provides supervision for the learning process. The unlabeled data helps the model capture additional patterns and improve generalization. This strategy improves performance on unseen data and reduces reliance on costly labeled datasets. Most studies in the field use a similar ratio, making it a standard practice in semi-supervised learning.

Before training, all input images were processed through a standardized pipeline. This ensured consistency across datasets and helped improve model performance. First, all images were resized to 224 × 224 pixels to match the input dimensions required by the convolutional backbone. Next, pixel values were normalized to the [0, 1] range. A color normalization step was also applied to reduce lighting and contrast variability across different acquisition devices. This step improves the ability of the model to generalize across diverse imaging conditions.

The HAM10000 dataset frequently contains occlusions such as hair artifacts and ruler marks. To address this issue, we applied the DullRazor algorithm. This technique detects and removes linear structures that resemble hair using morphological closing and bilinear interpolation. At the same time, it preserves important lesion details. This process reduces noise and improves lesion visibility, enhancing feature extraction during model training.

To address the class imbalance, especially in the ISIC-2020 dataset, where melanoma cases are rare, we used a hybrid strategy. This strategy combines random oversampling of the minority class with on-the-fly data augmentation. The augmentations include horizontal and vertical flips, random zooms, and slight rotations. These transformations increase diversity within underrepresented classes and reduce model bias during training. This approach ensures that the model is sufficiently exposed to all classes during training. As a result, it improves the sensitivity and robustness of the model in detecting malignant lesions.

### Metrics

This article uses Accuracy, F-measure, G-means, and AUC to evaluate the proposed model. These metrics were chosen as they provide a comprehensive assessment of performance across various aspects. Accuracy measures the proportion of correct predictions but can be misleading on imbalanced datasets. F-measure, the harmonic mean of precision and recall, is useful when one class is underrepresented. G-means, the geometric mean of sensitivity and specificity, evaluates balanced performance across classes, making it suitable for imbalanced datasets. AUC, based on the ROC curve, measures the discriminative power of the model across thresholds, offering a robust and class-independent performance indicator.

In addition to these technical metrics, we use clinically significant measures, including true positive rate (TPR) and false negative rate (FNR). These metrics are especially crucial in high-risk domains such as melanoma diagnosis. TPR, also known as sensitivity or recall, measures the proportion of actual positive cases that the model correctly identifies. This is a clinically critical metric in melanoma detection, where missing a malignant case can be fatal. In such cases, a high TPR helps ensure that the model correctly identifies patients with melanoma. This makes it highly valuable in clinical decision-making. On the other hand, FNR quantifies the proportion of actual positive cases that are incorrectly classified as negative. A high FNR means the model misses many melanoma cases. This failure may result in delayed diagnosis and treatment. Such outcomes have serious consequences in real-world medical settings. As such, minimizing the FNR is crucial for ensuring patient safety.

Mathematically, Accuracy, F-measure, G-means, TPR, and FNR are defined as follows:

17 $$\:\text{A}\text{c}\text{c}\text{u}\text{r}\text{a}\text{c}\text{y}=\frac{TP+TN}{Total\:EquationNumber\:of\:samples}$$

18 $$\:\text{F}-\text{m}\text{e}\text{a}\text{s}\text{u}\text{r}\text{e}=2\times\:\frac{Precision\times\:Recall}{Precision+Recall}$$

19 $$\:G-means=\sqrt{Recall\times\:\:Specificity}$$

20 $$\:\text{T}\text{P}\text{R}=\text{R}\text{e}\text{c}\text{a}\text{l}\text{l}=\frac{TP}{TP+FN}$$

21 $$\:\text{F}\text{N}\text{R}=1-\text{F}\text{N}\text{R}$$

where

22 $$\:\text{P}\text{r}\text{e}\text{c}\text{i}\text{s}\text{i}\text{o}\text{n}=\frac{TP}{TP+FP}$$

23 $$\:Specificity=\frac{TN}{TN+FP}$$

In this context, TP are cases where the model accurately identifies positive outcomes, and true negatives (TN) are cases where the model correctly predicts negative outcomes. False positives (FP) arise when the model incorrectly classifies negative instances as positive, and FN occur when the model overlooks positive instances.

### Model performance

The study was conducted on a 64-bit Windows operating system using high-performance hardware that included an Intel Core i9 processor and an NVIDIA GeForce RTX 3080 GPU, which provided the computational power necessary to process large datasets and complex neural network architectures efficiently. The research utilized Python programming language with TensorFlow and PyTorch frameworks, which facilitated the development of the SS-GAN and the implementation of the improved ABC algorithm for hyperparameter tuning. The software setup was chosen to ensure robustness and speed in model training and evaluation. TensorFlow and PyTorch were selected for their comprehensive libraries, ease of use, and strong community support, which are crucial for implementing cutting-edge machine-learning techniques. GPU acceleration was critical for handling the computationally intensive tasks of training deep neural networks and processing high-resolution dermoscopic images from the ISIC-2020, HAM10000, and PH2 datasets.

**Table 4:** Performance comparison of ML, DL, TL, ablation, and proposed models on the ISIC-2020 dataset.

Category	Model name	Accuracy	F-measure	G-means	AUC	TPR	FNR
	MPMRF^24^	67.511 ± 0.057	70.202 ± 0.051	71.929 ± 0.095	0.711 ± 0.076	70.511 ± 0.089	29.489 ± 0.041
Machine leaning	BASCA-Opt^25^	69.102 ± 0.056	73.237 ± 0.037	73.879 ± 0.098	0.749 ± 0.027	73.325 ± 0.048	26.675 ± 0.004
	CFBD-CM^26^	67.289 ± 0.039	71.493 ± 0.019	72.257 ± 0.041	0.731 ± 0.074	71.521 ± 0.039	28.479 ± 0.099
	NIHSC-System^27^	68.390 ± 0.042	72.967 ± 0.044	73.687 ± 0.067	0.737 ± 0.000	72.022 ± 0.012	27.978 ± 0.015
	KNN-HFIP^28^	68.572 ± 0.012	72.982 ± 0.055	73.147 ± 0.098	0.742 ± 0.016	73.026 ± 0.071	26.974 ± 0.024
Deep learning	WDNR^6^	68.142 ± 0.012	73.357 ± 0.056	73.985 ± 0.014	0.749 ± 0.016	73.556 ± 0.065	26.444 ± 0.023
	ECSD-Net^29^	69.364 ± 0.005	73.966 ± 0.089	74.683 ± 0.082	0.745 ± 0.083	74.256 ± 0.054	25.744 ± 0.093
	DDCNN-F^33^	80.877 ± 0.099	83.949 ± 0.023	84.456 ± 0.006	0.782 ± 0.059	83.999 ± 0.001	16.001 ± 0.017
	YOLOv7-XAI^34^	82.687 ± 0.041	84.774 ± 0.078	85.313 ± 0.079	0.802 ± 0.027	84.852 ± 0.037	15.148 ± 0.090
	MRFO-SCC^37^	85.499 ± 0.089	87.284 ± 0.079	88.852 ± 0.061	0.802 ± 0.087	87.356 ± 0.038	12.644 ± 0.016
	SSGNet^38^	82.575 ± 0.021	84.400 ± 0.057	86.024 ± 0.073	0.795 ± 0.024	85.125 ± 0.065	14.875 ± 0.056
	FixMatch-LS^39^	79.790 ± 0.030	80.081 ± 0.039	82.688 ± 0.005	0.783 ± 0.069	81.365 ± 0.097	18.635 ± 0.099
	NCPLSL^40^	84.243 ± 0.064	86.112 ± 0.082	87.662 ± 0.061	0.805 ± 0.083	86.355 ± 0.000	13.645 ± 0.026
	FaxMatch^13^	85.786 ± 0.040	86.392 ± 0.031	87.948 ± 0.008	0.812 ± 0.005	86.426 ± 0.085	13.574 ± 0.073
	GANA-SFE^41^	84.262 ± 0.046	85.445 ± 0.062	86.076 ± 0.081	0.818 ± 0.099	85.625 ± 0.099	14.375 ± 0.039
	STFL^42^	83.041 ± 0.040	86.425 ± 0.062	87.855 ± 0.058	0.836 ± 0.010	86.572 ± 0.020	13.428 ± 0.066
	DL-AMC^44^	70.341 ± 0.053	75.256 ± 0.037	75.883 ± 0.091	0.753 ± 0.043	75.362 ± 0.064	24.638 ± 0.047
	ODLA-Net^45^	71.481 ± 0.018	76.921 ± 0.087	77.521 ± 0.043	0.759 ± 0.057	76.996 ± 0.061	23.004 ± 0.036
	VGG-16-GAN^46^	82.369 ± 0.074	83.405 ± 0.099	84.944 ± 0.072	0.802 ± 0.031	83.823 ± 0.003	16.177 ± 0.059
	GANViT-MD^47^	79.681 ± 0.054	82.147 ± 0.064	83.803 ± 0.011	0.806 ± 0.011	82.584 ± 0.095	17.416 ± 0.069
	PPO-GAN-MC^48^	87.861 ± 0.064	88.150 ± 0.046	89.779 ± 0.060	0.822 ± 0.020	88.778 ± 0.078	11.222 ± 0.015
Transfer learning	HE-TLMC^51^	85.459 ± 0.089	87.734 ± 0.009	88.169 ± 0.098	0.840 ± 0.002	87.402 ± 0.051	12.598 ± 0.009
	DLCA-SC^55^	87.209 ± 0.027	88.481 ± 0.083	89.971 ± 0.028	0.845 ± 0.073	88.575 ± 0.077	11.425 ± 0.082
	QDLM-NM^56^	75.874 ± 0.044	82.237 ± 0.010	82.754 ± 0.096	0.807 ± 0.068	82.365 ± 0.003	17.635 ± 0.008
	WE-TLMC^59^	72.883 ± 0.092	80.267 ± 0.083	80.855 ± 0.035	0.777 ± 0.065	80.369 ± 0.059	19.631 ± 0.092
	XAI-MRA^61^	85.580 ± 0.093	86.215 ± 0.021	89.759 ± 0.049	0.833 ± 0.010	87.380 ± 0.033	12.620 ± 0.077
	TL-MD^62^	74.243 ± 0.068	81.173 ± 0.014	81.718 ± 0.062	0.792 ± 0.022	81.314 ± 0.037	18.686 ± 0.022
Ablation	Proposed w/o RL	80.404 ± 0.018	82.241 ± 0.074	83.812 ± 0.056	0.782 ± 0.094	82.325 ± 0.021	17.675 ± 0.062
	Proposed w/o SA	84.180 ± 0.073	86.524 ± 0.050	87.056 ± 0.051	0.805 ± 0.082	86.624 ± 0.063	13.376 ± 0.009
	Proposed w/o CR	86.124 ± 0.003	87.657 ± 0.053	88.148 ± 0.078	0.816 ± 0.047	87.775 ± 0.054	12.225 ± 0.026
	Proposed w/o PL	81.134 ± 0.009	83.750 ± 0.006	85.279 ± 0.026	0.793 ± 0.029	84.230 ± 0.074	15.770 ± 0.011
	Proposed w/o HO	82.711 ± 0.011	84.782 ± 0.008	85.309 ± 0.059	0.796 ± 0.044	84.865 ± 0.062	15.135 ± 0.084
Proposed	Proposed	90.517 ± 0.005	92.769 ± 0.021	93.508 ± 0.016	0.893 ± 0.026	92.825 ± 0.027	7.175 ± 0.061

**Table 5:** Performance comparison of ML, DL, TL, ablation, and proposed models on the HAM10000 dataset.

Category	Model name	Accuracy	F-measure	G-means	AUC	TPR	FNR
	MPMRF^24^	69.511 ± 0.047	72.202 ± 0.030	73.929 ± 0.026	0.722 ± 0.079	72.511 ± 0.060	27.489 ± 0.071
Machine leaning	BASCA-Opt^25^	70.182 ± 0.025	73.450 ± 0.019	75.200 ± 0.004	0.758 ± 0.019	74.149 ± 0.071	25.851 ± 0.015
	CFBD-CM^26^	70.151 ± 0.021	74.419 ± 0.006	75.169 ± 0.001	0.740 ± 0.059	74.681 ± 0.086	25.319 ± 0.099
	NIHSC-System^27^	71.098 ± 0.077	75.027 ± 0.024	75.785 ± 0.093	0.750 ± 0.074	75.242 ± 0.020	24.758 ± 0.082
	KNN-HFIP^28^	71.511 ± 0.025	72.202 ± 0.083	74.929 ± 0.017	0.762 ± 0.076	73.261 ± 0.003	26.739 ± 0.005
Deep learning	WDNR^6^	69.890 ± 0.057	74.995 ± 0.033	75.636 ± 0.095	0.862 ± 0.028	74.998 ± 0.094	25.002 ± 0.029
	ECSD-Net^29^	72.068 ± 0.080	76.088 ± 0.029	76.868 ± 0.061	0.757 ± 0.059	76.273 ± 0.046	23.727 ± 0.009
	DDCNN-F^33^	81.511 ± 0.066	84.202 ± 0.031	86.929 ± 0.036	0.790 ± 0.091	85.884 ± 0.058	14.116 ± 0.036
	YOLOv7-XAI^34^	83.207 ± 0.002	85.464 ± 0.065	86.205 ± 0.092	0.805 ± 0.047	85.575 ± 0.077	14.425 ± 0.060
	MRFO-SCC^37^	86.768 ± 0.021	88.187 ± 0.007	89.945 ± 0.073	0.812 ± 0.004	88.232 ± 0.004	11.768 ± 0.002
	SSGNet^38^	83.388 ± 0.098	85.227 ± 0.057	87.919 ± 0.027	0.801 ± 0.032	86.111 ± 0.045	13.889 ± 0.099
	FixMatch-LS^39^	80.150 ± 0.025	82.857 ± 0.084	84.527 ± 0.089	0.792 ± 0.096	83.606 ± 0.030	16.394 ± 0.086
	NCPLSL^40^	86.702 ± 0.049	87.164 ± 0.071	88.760 ± 0.029	0.813 ± 0.056	87.384 ± 0.018	12.616 ± 0.016
	FaxMatch^13^	87.955 ± 0.022	88.197 ± 0.006	89.757 ± 0.036	0.822 ± 0.006	88.456 ± 0.063	11.544 ± 0.038
	GANA-SFE^41^	86.779 ± 0.049	87.763 ± 0.097	89.319 ± 0.081	0.831 ± 0.088	88.252 ± 0.019	11.748 ± 0.026
	STFL^42^	84.538 ± 0.018	86.064 ± 0.023	88.653 ± 0.039	0.843 ± 0.067	87.339 ± 0.081	12.661 ± 0.078
	DL-AMC^44^	72.678 ± 0.085	76.681 ± 0.026	77.457 ± 0.008	0.770 ± 0.002	76.389 ± 0.097	23.611 ± 0.029
	ODLA-Net^45^	73.380 ± 0.062	77.403 ± 0.012	78.204 ± 0.049	0.782 ± 0.044	77.645 ± 0.009	22.355 ± 0.034
	VGG-16-GAN^46^	83.245 ± 0.012	84.933 ± 0.058	85.448 ± 0.036	0.809 ± 0.010	85.005 ± 0.009	14.995 ± 0.080
	GANViT-MD^47^	80.238 ± 0.092	83.090 ± 0.045	85.665 ± 0.008	0.828 ± 0.096	83.610 ± 0.079	16.390 ± 0.012
	PPO-GAN-MC^48^	88.788 ± 0.046	89.321 ± 0.035	90.832 ± 0.007	0.936 ± 0.054	89.419 ± 0.092	10.581 ± 0.038
Transfer learning	HE-TLMC^51^	87.491 ± 0.016	88.193 ± 0.009	89.710 ± 0.062	0.846 ± 0.063	88.202 ± 0.033	11.798 ± 0.027
	DLCA-SC^55^	89.244 ± 0.071	90.402 ± 0.068	90.888 ± 0.002	0.858 ± 0.067	90.549 ± 0.058	9.451 ± 0.038
	QDLM-NM^56^	78.169 ± 0.017	82.257 ± 0.062	83.004 ± 0.050	0.826 ± 0.026	82.369 ± 0.035	17.631 ± 0.041
	WE-TLMC^59^	75.666 ± 0.075	80.041 ± 0.068	80.791 ± 0.068	0.806 ± 0.075	80.205 ± 0.033	19.795 ± 0.019
	XAI-MRA^61^	86.021 ± 0.005	87.019 ± 0.040	88.514 ± 0.042	0.855 ± 0.051	87.396 ± 0.017	12.604 ± 0.037
	TL-MD^62^	77.431 ± 0.020	81.457 ± 0.053	82.162 ± 0.068	0.821 ± 0.093	81.533 ± 0.018	18.467 ± 0.098
Ablation	Proposed w/o RL	82.893 ± 0.090	83.383 ± 0.041	85.116 ± 0.040	0.798 ± 0.015	84.130 ± 0.060	15.870 ± 0.085
	Proposed w/o SA	86.535 ± 0.049	88.430 ± 0.024	89.886 ± 0.017	0.812 ± 0.033	88.602 ± 0.084	11.398 ± 0.098
	Proposed w/o CR	87.999 ± 0.045	89.743 ± 0.015	90.191 ± 0.043	0.825 ± 0.003	89.800 ± 0.016	10.200 ± 0.051
	Proposed w/o PL	82.562 ± 0.039	85.019 ± 0.047	86.488 ± 0.037	0.809 ± 0.041	85.104 ± 0.084	14.896 ± 0.060
	Proposed w/o HO	84.554 ± 0.067	86.500 ± 0.011	88.175 ± 0.008	0.815 ± 0.081	87.052 ± 0.028	12.948 ± 0.059
Proposed	Proposed	92.358 ± 0.086	93.376 ± 0.074	94.500 ± 0.051	0.902 ± 0.020	93.482 ± 0.049	6.518 ± 0.032

We used 5-fold stratified cross-validation to evaluate the robustness and accuracy of our model on the ISIC-2020 and HAM10000 datasets. This method ensures that each fold reflects the same class distribution as the entire dataset. This is particularly important for imbalanced cases like melanoma detection, where positive cases are much fewer than negative ones. Stratification reduces model bias toward the majority class. It also improves generalization by ensuring that both melanoma and non-melanoma cases are well represented in each fold. In each round, four folds are used for training and one for testing, with all samples eventually serving as test data. This approach provides a comprehensive performance evaluation across the dataset and reduces result variance. Notably, the PH2 and DermNet datasets were used solely for external testing, not for training or validation. All reported results are presented as mean ± standard deviation ($\:M\:\pm\:\:\sigma\:$) to reflect consistency and reliability across folds.

During the evaluation phase, the proposed model was compared against a range of baseline methods. These included five machine learning models: MPMRF (multi-phase melanoma recognition framework)^24^ BASCA-Opt (bat-algorithm-based skin cancer analysis optimizer)^25^ CFBD-CM (counting fractal box dimension classification method)^26^ NIHSC-System (near-infrared hyperspectral signal classification system)^27^ KNN-HFIP (k-nearest neighbors hybrid-fused indoor positioning approach)^28^.

In addition, sixteen deep learning approaches were considered: WDNR (Wavelet-based classification through deep neural architectures)^6^ECSD-Net (ensemble classification for skin disease detection network)^29^ DDCNN-F (double decker CNN ‘F’ feature fusion)^33^ YOLOv7-XAI^34^ MRFO-SCC (manta ray foraging optimizer for skin cancer classification)^37^ SSGNet (semi-supervised multi-path grid network for diagnosing melanoma)^38^FixMatch-LS (semi-supervised skin lesion classification with label smoothing)^39^ NCPLSL (noisy-consistent pseudo labeling model for semi-supervised)^40^FaxMatch (multi-curriculum pseudo‐labeling for semi‐supervised medical image classification)^13^GANA-SFE (GAN-based augmentation and self-supervised feature extractor for melanoma)^41^ STFL (self-feedback threshold focal learning)^42^ DL-AMC (deep learning-based automated melanoma classification)^44^ ODLA-Net (optimized deep learning architecture for skin lesion analysis)^45^ VGG-16-GAN^46^ GANViT-MD (melanoma detection via GAN synthesis and vision transformer)^47^ PPO-GAN-MC (proximal policy optimized GAN for melanoma classification)^48^.

Furthermore, six transfer learning models were included in the comparison: HE-TLMC (hybrid ensemble transfer learning melanoma classifier)^51^ DLCA-SC (deep learning comprehensive analysis for skin cancer)^55^ QDLM-NM (quantized deep learning model for nail melanoma)^56^ WE-TLMC (weighted ensemble-based transfer learning for melanoma classification)^59^ XAI-MRA (explainable AI model for melanoma risk assessment)^61^ TL-MD (transfer learning-enhanced melanoma detection)^62^.

Finally, ablation studies were conducted by comparing the proposed model with five of its derivatives: Proposed w/o RL, w/o SA, w/o CR, w/o PL, and w/o HO. These versions exclude, respectively, reconstruction loss (RL), self-attention (SA), consistency regularization (CR), pseudo-labeling (PL), and hyperparameter optimization (HO) in the SS-GAN framework.

The results of these experiments for ISIC-2020 and HAM10000 datasets are summarized in Tables 4 and 5. An analysis of state-of-the-art models on ISIC-2020 and HAM10000 datasets shows that traditional ML methods underperform. They are less effective than DL and TL approaches. ML models, such as MPMRF, BASCA-Opt, and CFBD-CM, exhibit limited feature representation and poor handling of class imbalance. Their TPR values remain below 75%, with FNRs ranging from 25% to nearly 30%, indicating a weak sensitivity to melanoma. DL models offer notable improvements. MRFO-SCC, NCPLSL, and FaxMatch outperform ECSD-Net and FixMatch-LS in TPR by 10–15% and also significantly reduce FNRs. However, models like DL-AMC and ODLA-Net still struggle due to a lack of self-supervision or inadequate augmentation. TL models, such as DLCA-SC and HE-TLMC, consistently achieve TPR above 88%, benefiting from pre-trained extractors and ensemble techniques. While DL outperforms ML, TL provides additional gains through ensemble learning and explainability. However, most models still struggle to generalize to unlabeled data and remain sensitive to hyperparameter tuning in real-world diagnosis.

Across both datasets, the proposed SS-GAN outperforms all models from traditional ML, DL, and TL categories. On ISIC-2020, SS-GAN achieves a TPR of 92.825%, which is 6–12% higher than top semi-supervised models, such as STFL, FaxMatch, and GANViT-MD. Compared to the best DL model (PPO-GAN-MC), SS-GAN improves the TPR by approximately 4% and the F-measure by over 4.5%. Relative to the strongest TL model (DLCA-SC), it achieves a 4.2% increase in TPR and a 4.3% increase in F-measure. Against BASCA-Opt, it outperforms by over 19% in both metrics. On HAM10000, SS-GAN maintains an edge, with a TPR 3–10% higher than that of semi-supervised competitors and 4.9% higher than PPO-GAN-MC. It also surpasses DLCA-SC and BASCA-Opt by 2.9–13% across metrics. These results stem from the use of reconstruction loss to reduce mode collapse, self-attention for handling long-range dependencies, consistency regularization, confidence-based pseudo-labeling, and hyperparameter optimization, collectively improving feature generalization, stability, and classification fidelity.

The ablation results provide a clear justification for the architectural choices made in the proposed SS-GAN model. Each component contributes uniquely to the final performance. Removing RL results in a significant drop of over 9% in TPR and G-means for both ISIC-2020 and HAM10000. This shows its role in preventing mode collapse and maintaining latent feature consistency. Excluding SA reduces the modeling of long-range dependencies. This is reflected by a 4–6% decrease in TPR in ISIC-2020 and a slightly smaller drop in HAM10000. Omitting CR decreases stability in the presence of input perturbations. This results in a 3–5% performance drop, particularly evident in the ISIC-2020 dataset. The PL mechanism enhances the use of unlabeled data by filtering low-confidence predictions; its absence leads to TPR and AUC drops of up to 8% in HAM10000. HO ensures convergence to optimal configurations; without it, the model underperforms by 6–7% across key metrics in both datasets. Notably, prior semi-supervised models such as FixMatch-LS and GANViT-MD did not integrate all these components, particularly lacking CR and HO strategies. Therefore, the combined effect of all architectural elements in SS-GAN explains its superior generalization, robustness, and accuracy across diverse imaging scenarios.

Two-tailed paired t-tests were performed on six metrics (Accuracy, F-measure, G-means, AUC, TPR, and FNR) to validate the statistical significance of the improvements, comparing SS-GAN with the top models. On ISIC-2020, SS-GAN significantly outperformed BASCA-Opt with p-values of 0.0004, 0.0006, 0.0002, 0.0009, 0.0011, and 0.0013, respectively. Against PPO-GAN-MC, values ranged from 0.0015 to 0.0026, while for DLCA-SC, they were between 0.0019 and 0.0042. For semi-supervised models like FaxMatch and STFL, p-values were all below 0.003. On HAM10000, p-values compared to BASCA-Opt were 0.0003 to 0.0012, versus PPO-GAN-MC 0.0021 to 0.0040, and versus DLCA-SC 0.0028 to 0.0046. Overall, comparisons between the proposed model and other state-of-the-art models across both datasets show consistently low p-values. 95% confidence intervals remained narrow (± 0.01 to ± 0.05) across both datasets. These findings show that the proposed model surpasses existing models in performance metrics. It achieves this with high statistical significance, ensuring that the observed improvements are both authentic and meaningful.

Table 6 Provides an analysis of the computational efficiency of various models. It assesses runtime and GPU usage across the ISIC-2020 and HAM10000 datasets. The proposed SS-GAN exhibits favorable runtime (3042s on ISIC-2020, 2698 s on HAM10000) and moderate GPU usage (21.2 GB, 18.2 GB). Compared to semi-supervised baselines like FixMatch-LS and faxmatch, SS-GAN improves runtime by 8–18%. It also reduces GPU usage by up to 35%, averaged across both datasets. Furthermore, SS-GAN is more efficient than the top DL model, PPO-GAN-MC (3512s, 28.5GB on ISIC-2020). It also surpasses the best TL model, DLCA-SC (3267s, 28.4GB on ISIC-2020). On ISIC-2020, SS-GAN reduces runtime by 13.4% compared to PPO-GAN-MC and by 2.8% compared to DLCA-SC. It also lowers GPU usage by 25.6% and 25.4%, respectively. Similar trends are observed on HAM10000. These findings validate SS-GAN as a scalable, resource-efficient solution well-suited for clinical deployment.

**Table 6:** Comparative analysis of runtime and GPU usage for various models on the ISIC-2020 and HAM10000 datasets.

Model name	ISIC-2020		HAM10000	
	Runtime (s)	GPU usage (GB)	Runtime (s)	GPU usage (GB)
MPMRF^24^	2091	18.5	1945	17.4
BASCA-Opt^25^	2053	19.4	2012	17.7
CFBD-CM^26^	2841	18.2	2614	17.2
NIHSC-System^27^	2459	22.4	2145	18.3
KNN-HFIP^28^	2034	23.7	1982	21.6
WDNR^6^	2656	24.6	2433	22.9
ECSD-Net^29^	3041	18.7	2530	19.4
DDCNN-F^33^	2512	23.5	2463	21.2
YOLOv7-XAI^34^	2695	25.1	2536	24.9
MRFO-SCC^37^	3156	24.6	3025	23.1
SSGNet^38^	2965	27.2	2865	26.2
FixMatch-LS^39^	2956	28.6	2826	26.3
NCPLSL^40^	2435	25.8	2277	24.6
FaxMatch^13^	2749	28.6	2404	27.5
GANA-SFE^41^	2652	24.2	2579	22.3
STFL^42^	2807	27.1	2754	25.1
DL-AMC^44^	3256	23.9	2867	18.4
ODLA-Net^45^	3401	19.7	3206	17.9
VGG-16-GAN^46^	2963	25.3	2841	24.5
GANViT-MD^47^	2736	28.1	2507	23.3
PPO-GAN-MC^48^	3512	28.5	2837	20.9
HE-TLMC^51^	2831	23.6	2246	23.4
DLCA-SC^55^	3267	28.4	2507	25.6
QDLM-NM^56^	3418	24.2	3156	19.3
WE-TLMC^59^	3725	21.8	3469	19.3
XAI-MRA^61^	3377	27.0	3145	25.4
TL-MD^62^	3563	23.1	3203	20.7
Proposed	3042	21.2	2698	18.2

**Fig. 4:**
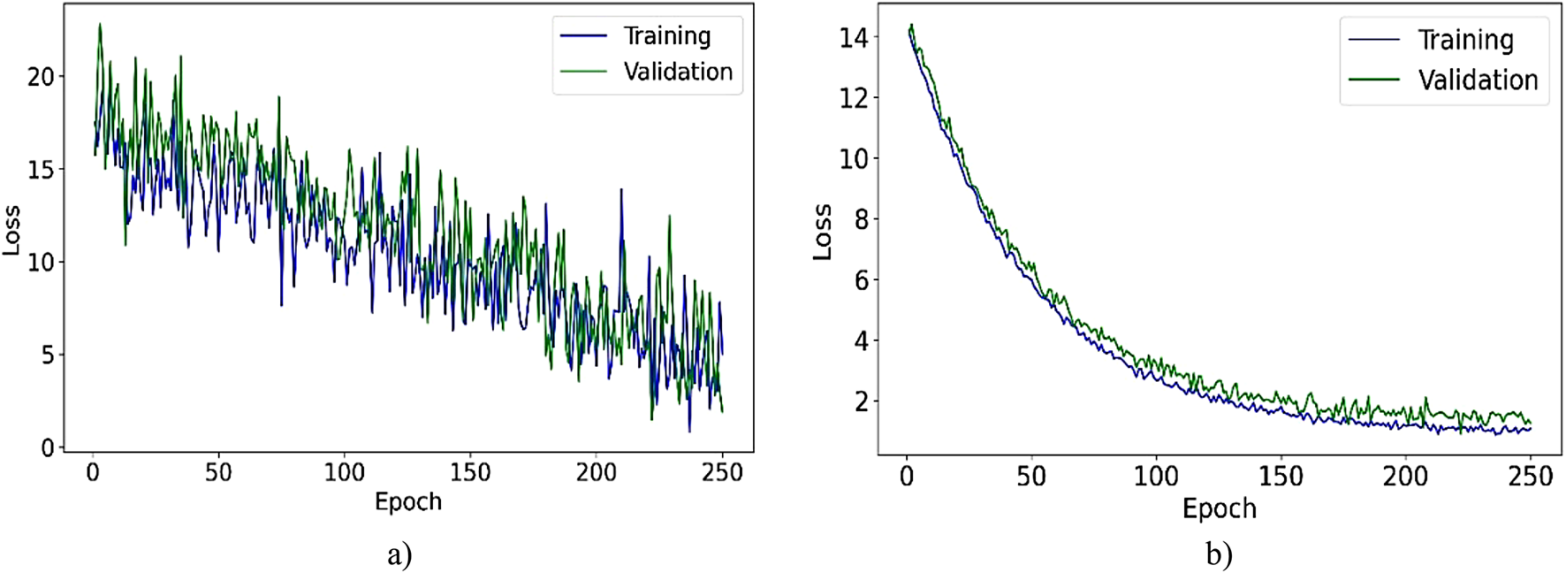
Training and validation loss curves over 250 epochs on the (**a**) ISIC-2020 and (**b**) HAM10000 datasets.

Figure 4shows the training and validation loss curves for 250 epochs on the ISIC-2020 and HAM10000 datasets. In both cases, the proposed model demonstrates stable convergence, with training and validation losses decreasing consistently. The minimal gap between the curves indicates strong generalization and the absence of overfitting. Especially on the HAM10000 dataset, the nearly overlapping loss curves highlight robust learning with minimal variance. On ISIC-2020, some fluctuations are present. However, the overall downward trend confirms effective convergence. These results validate the reliability and learning efficiency of the model across datasets.

**Fig. 5:**
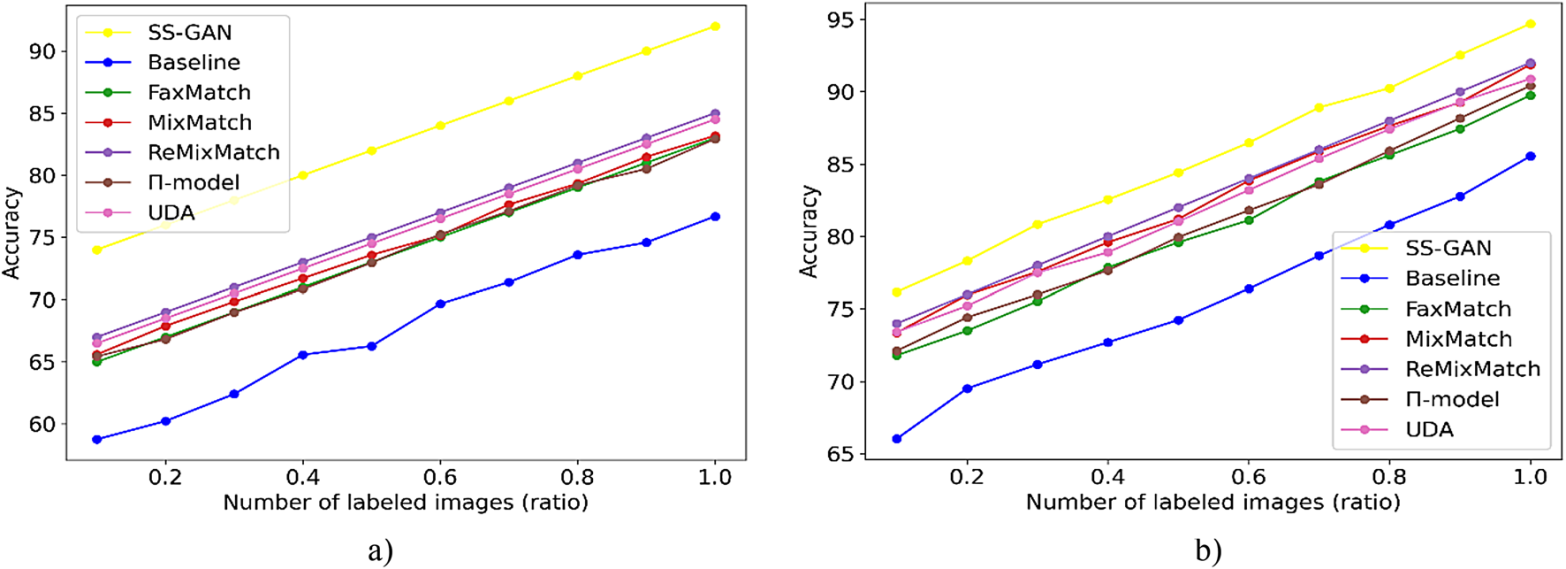
Accuracy trends for SS-GAN and baseline SSL models across various labeled samples for the (**a**) ISIC 2020 and (**b**) HAM10000 datasets.

Figure 5 illustrates the accuracy trends of the proposed SS-GAN in comparison with baseline SSL models, including FaxMatch (feature-based augmentation for semi-supervised learning), MixMatch (a comprehensive semi-supervised learning approach), ReMixMatch (improved MixMatch for semi-supervised learning), Π-model (pi-model), and UDA (unsupervised data augmentation). The comparison is performed on the ISIC-2020 and HAM10000 datasets across varying ratios of labeled data. SS-GAN consistently achieves superior accuracy, maintaining a 4–8% margin even with only 20% of the data labeled. This advantage stems from its architecture. Reconstruction loss mitigates mode collapse and increases data diversity, improving coverage of rare melanoma subtypes. Self-attention captures global spatial dependencies in dermoscopic images, ensuring critical features are not overlooked. Pseudo-labeling enhances the training set by incorporating high-confidence unlabeled samples, while consistency regularization stabilizes predictions in the presence of perturbations. Hyperparameter tuning with ML-ABC ensures optimal learning dynamics. It also prevents overfitting and achieves smooth convergence in challenging SSL settings. SS-GAN maintains its advantage as labeled data increases, proving scalability and adaptability. It outperforms other SSL methods by efficiently leveraging both labeled and unlabeled data. Its success comes from robust regularization and tuning. These mechanisms ensure consistent improvements and reliable performance, even under low-label conditions or varying labeling ratios.

**Fig. 6:**
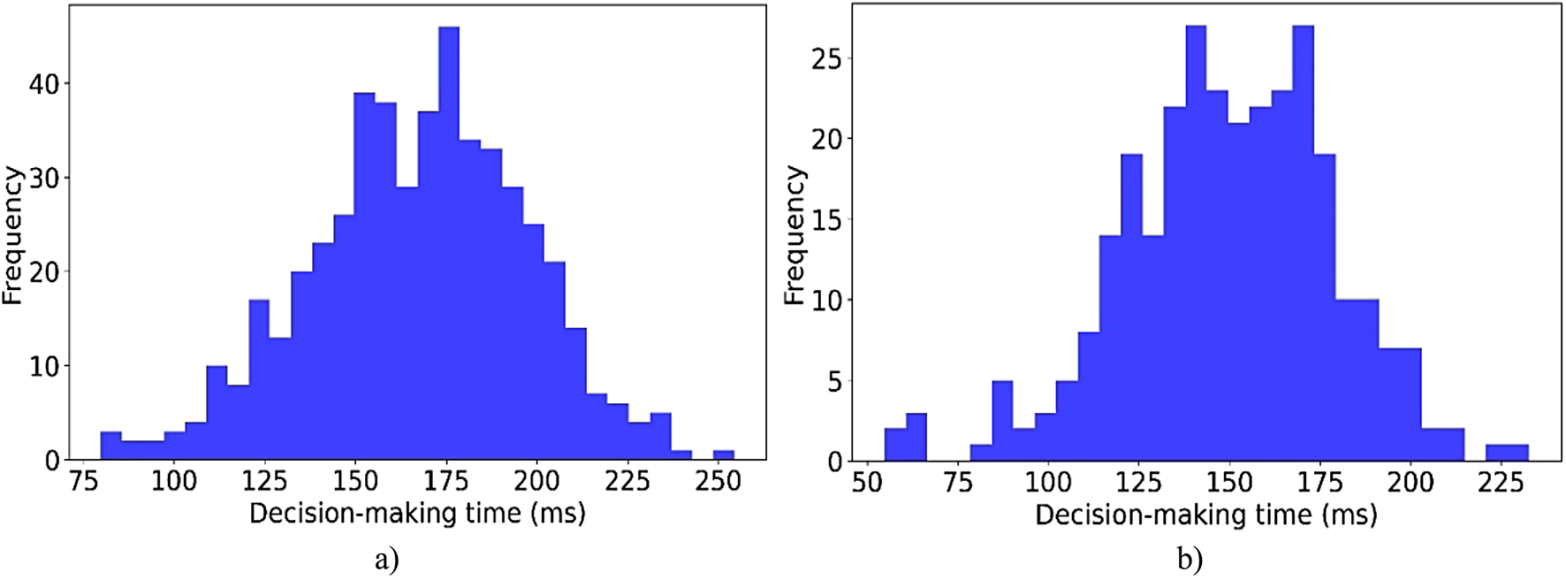
Real-time decision-making latency for the (a) ISIC-2020 and (b) HAM10000 datasets using the proposed model.

Figure 6 illustrates the decision-making time distributions of the proposed model in real-time settings, evaluated on the ISIC-2020 and HAM10000 datasets. The results show that most predictions were made within 150–190 ms for ISIC-2020. For HAM10000, prediction times ranged from 135 to 185 ms, confirming real-time suitability across both datasets. This demonstrates that the model consistently maintains low latency, a crucial requirement for real-time clinical settings. The tight distribution and low average inference time confirm the suitability of the mode for time-sensitive applications. It is well-suited for automated skin lesion screening, where rapid decision-making is critical for workflow efficiency.

**Fig. 7:**
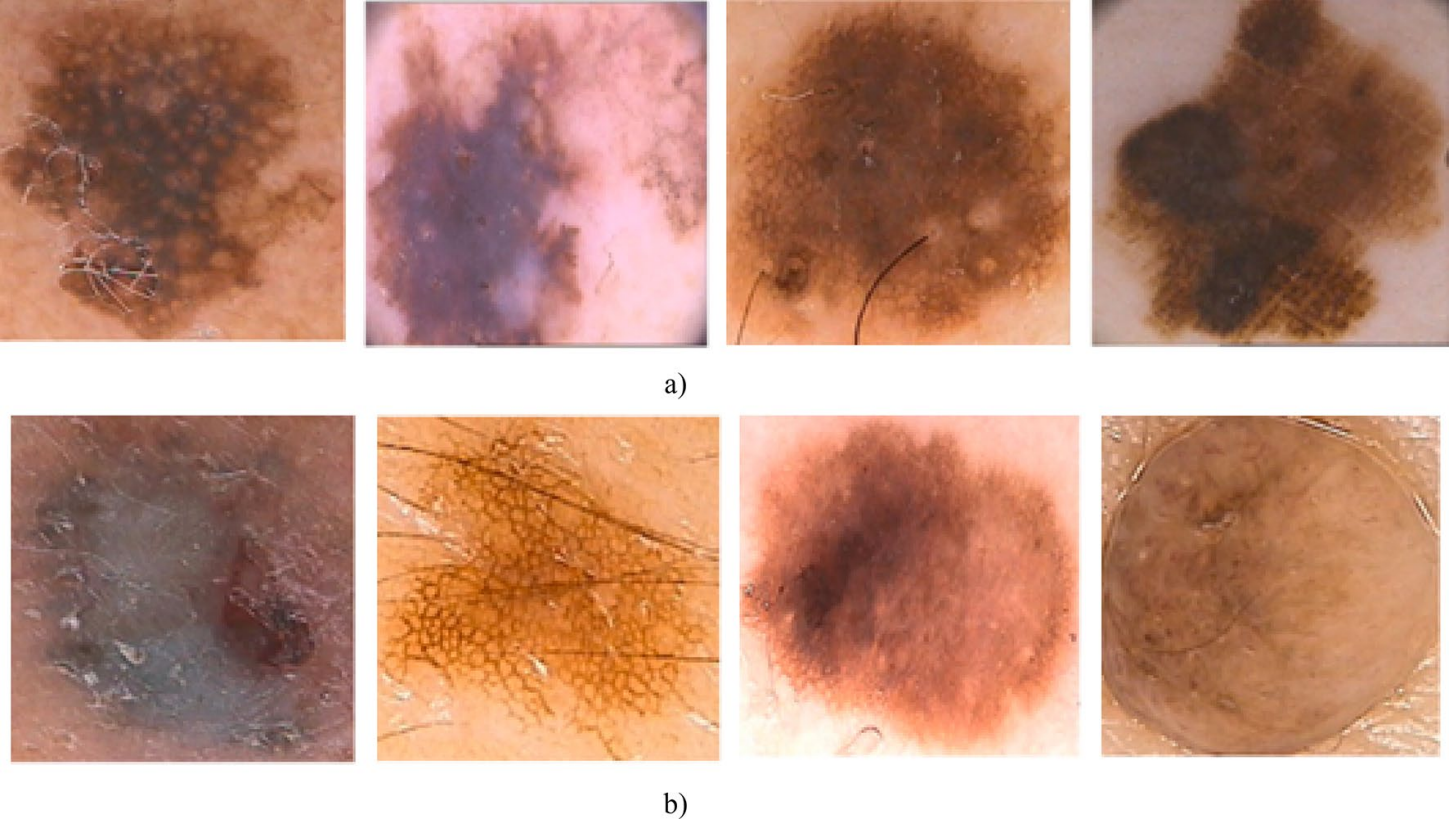
Misclassified samples in the HAM10000 dataset. a) Non-melanoma images incorrectly classified as melanoma. b) Melanoma images incorrectly classified as non-melanoma.

Figure 7 illustrates instances of misclassification by the proposed SS-GAN model in the HAM10000 dataset. These errors highlight the need for further refinement of classification models, especially for cases with subtle differences between melanoma and non-melanoma lesions. Despite the strong performance of the model, these misclassifications show where it struggles. Distinguishing rare melanoma cases from non-melanoma lesions becomes harder under complex imaging conditions. This analysis highlights the need for enhanced training to improve generalization across diverse datasets and effectively handle the nuances of skin lesion classification.

**Fig. 8:**
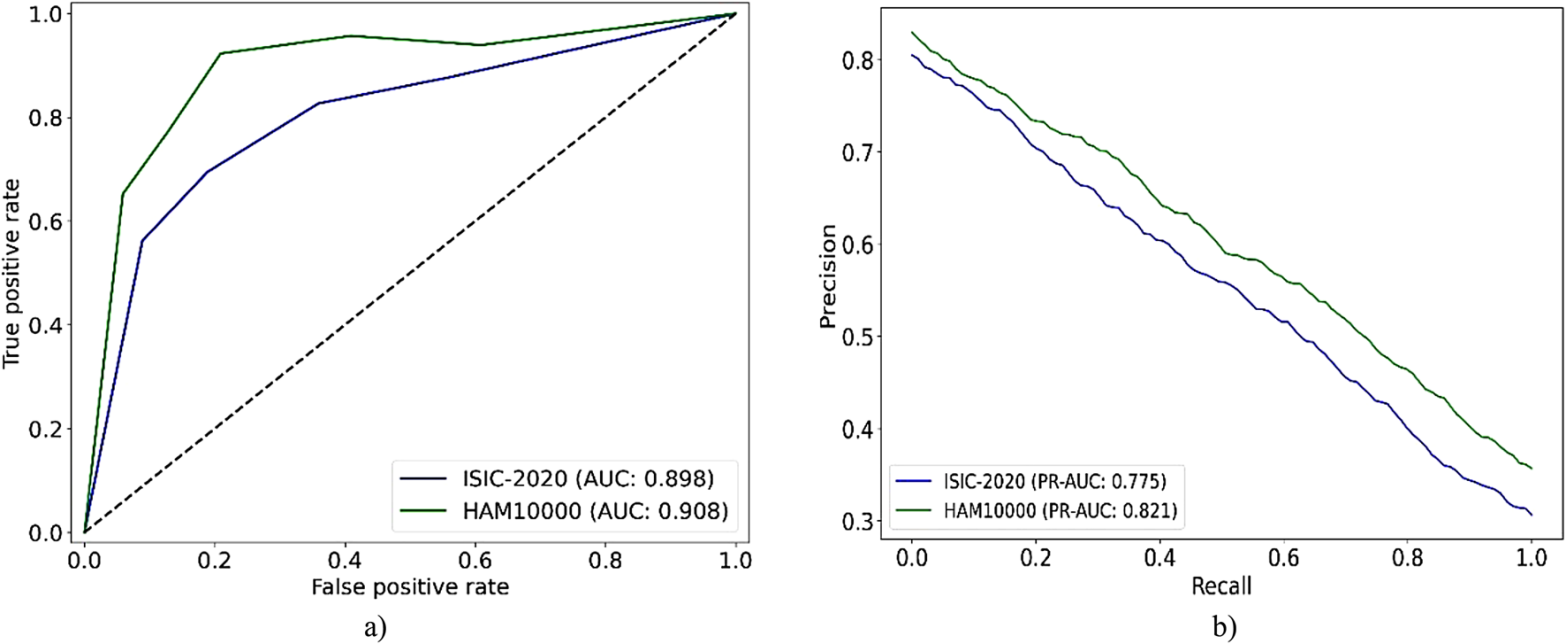
(a) ROC-AUC and (b) PR-AUC curves of the proposed model on the ISIC-2020 and HAM10000 datasets.

Figure 8shows the ROC and precision-recall (PR) curves of the proposed model for the ISIC-2020 and HAM10000 datasets. The AUC values of 0.898 (ISIC-2020) and 0.908 (HAM10000), along with PR-AUC scores of 0.775 and 0.821, respectively, indicate excellent discriminatory power. High PR-AUC values indicate that the model maintains strong precision across a wide range of recalls. This is critical for imbalanced classification problems. These curves confirm that the proposed model can reliably distinguish minority classes from dominant ones.

**Fig. 9:**
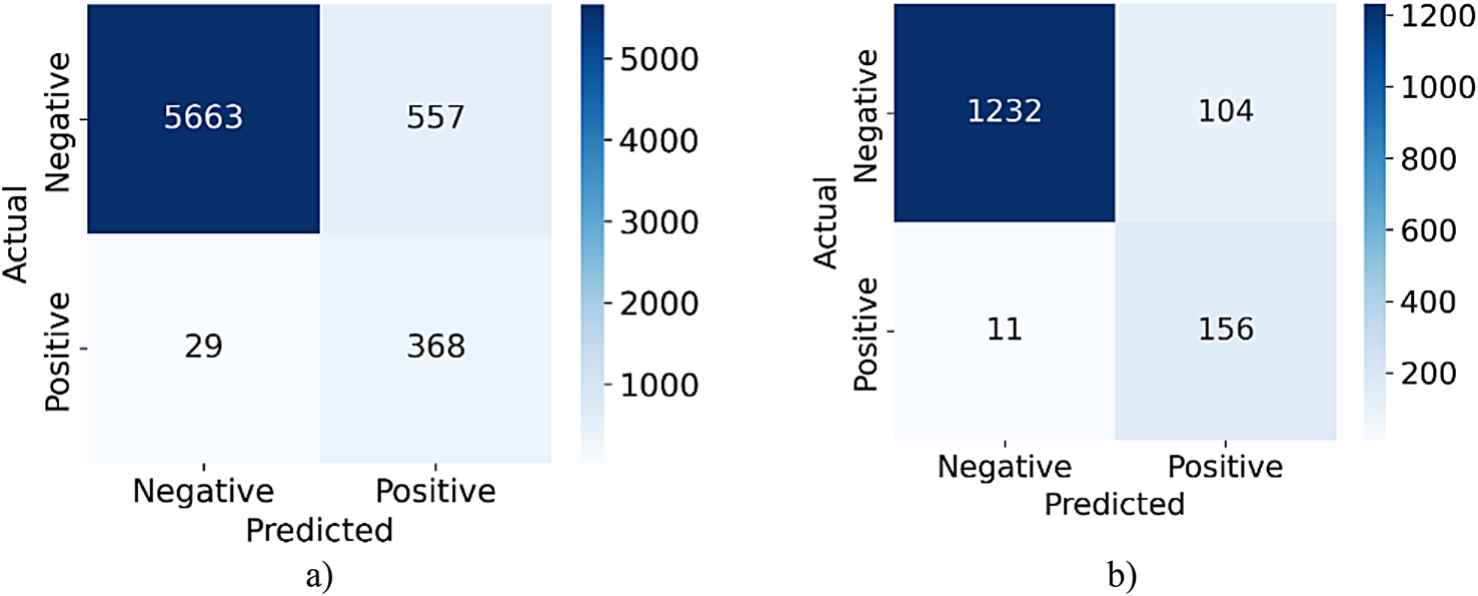
Confusion matrices of the proposed model on the (a) ISIC-2020 and (b) HAM10000 datasets.

Figure 9 presents the confusion matrices for the ISIC-2020 and HAM10000 datasets, demonstrating the strong performance of the proposed model. The model achieves a high number of TN and TP. It records only 29 FN and 557 FP for ISIC-2020, and 11 FN and 104 FP for HAM10000. These low FN and FP rates indicate that the model is reliable in identifying melanoma cases while minimizing misclassifications. This makes it highly suitable for diagnostic applications.

**Fig. 10:**
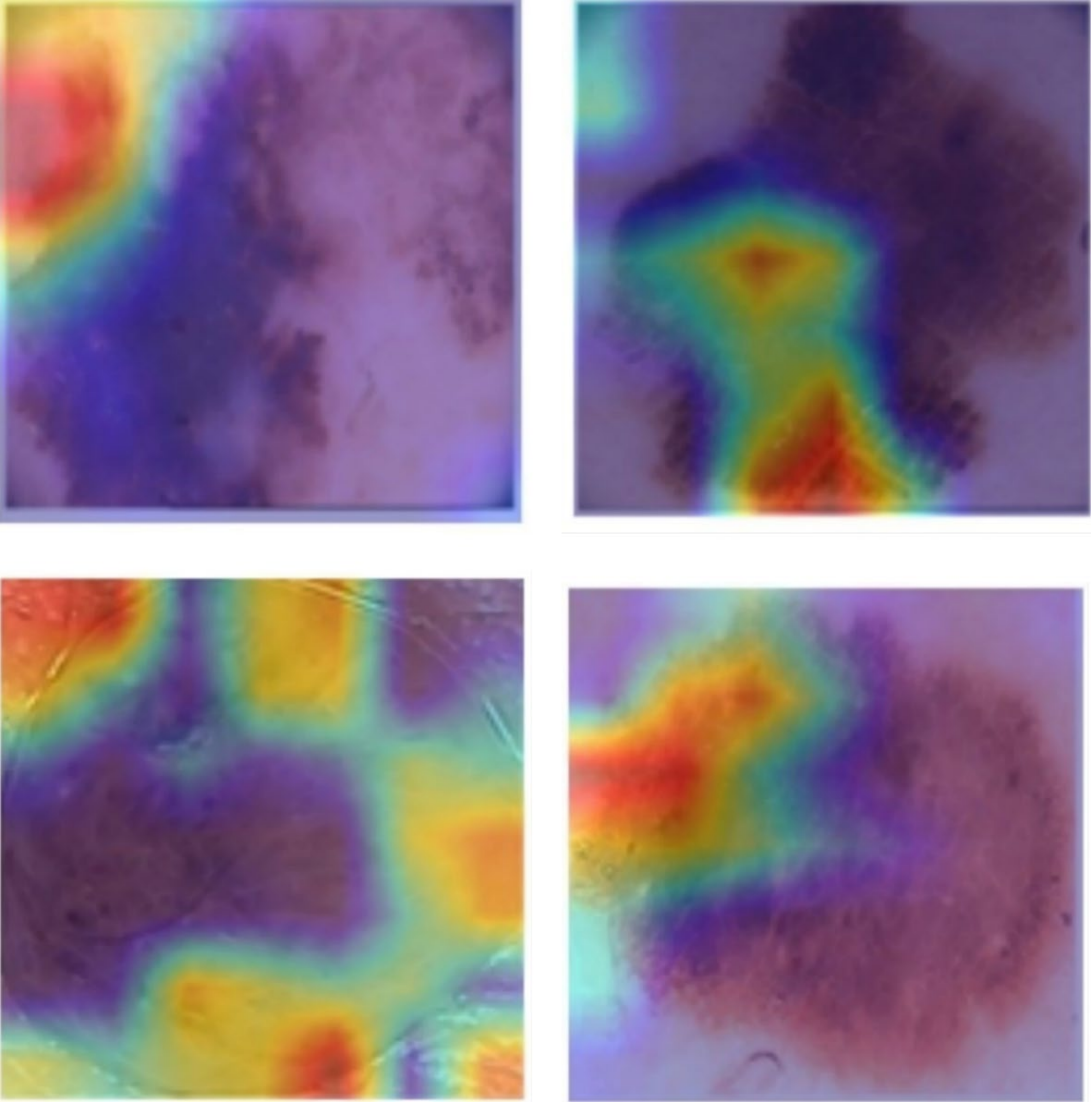
SHAP visualizations highlighting key regions influencing melanoma classification.

Figure 10 shows SHAP-based visualizations that highlight the key regions influencing the decisions of the model. In each image, areas shaded in red and yellow indicate features that significantly contribute to the final classification, while purple and blue signify regions that make a low contribution. Board-certified dermatologists have verified these high-importance areas as clinically relevant and linked to malignancy or atypical features. These visualizations demonstrate that the model consistently focuses on lesion boundaries and high-risk pigment zones, suggesting effective feature attribution. Consistent patterns across lesion types confirm the interpretability and transparency of the model. This supports its clinical reliability in dermatological diagnostics.

**Table 7:** Performance comparison of ML, DL, TL, ablation, and proposed models on the PH2 dataset.

Category	Model name	Accuracy	F-measure	G-means	AUC	TPR	FNR
	MPMRF^24^	61.511 ± 0.098	66.202 ± 0.036	67.929 ± 0.068	0.682 ± 0.017	67.066 ± 0.059	32.934 ± 0.066
Machine leaning	BASCA-Opt^25^	64.593 ± 0.034	69.717 ± 0.091	70.377 ± 0.092	0.710 ± 0.003	70.047 ± 0.095	29.953 ± 0.018
	CFBD-CM^26^	62.177 ± 0.078	67.596 ± 0.024	68.424 ± 0.040	0.707 ± 0.034	68.010 ± 0.025	31.990 ± 0.070
	NIHSC-System^27^	62.995 ± 0.070	68.326 ± 0.087	69.120 ± 0.053	0.714 ± 0.009	68.723 ± 0.064	31.277 ± 0.048
	KNN-HFIP^28^	63.403 ± 0.098	69.984 ± 0.061	69.659 ± 0.025	0.719 ± 0.010	69.822 ± 0.095	30.178 ± 0.025
Deep learning	WDNR^6^	63.333 ± 0.057	68.473 ± 0.024	70.129 ± 0.029	0.731 ± 0.041	79.469 ± 0.096	20.531 ± 0.067
	ECSD-Net^29^	64.538 ± 0.057	69.738 ± 0.012	70.528 ± 0.027	0.730 ± 0.049	70.133 ± 0.092	29.867 ± 0.019
	DDCNN-F^33^	73.511 ± 0.063	79.202 ± 0.021	80.929 ± 0.019	0.722 ± 0.003	79.562 ± 0.047	20.438 ± 0.097
	YOLOv7-XAI^34^	75.589 ± 0.006	80.396 ± 0.002	81.125 ± 0.097	0.743 ± 0.039	80.416 ± 0.064	19.584 ± 0.098
	MRFO-SCC^37^	80.835 ± 0.023	80.705 ± 0.059	81.409 ± 0.047	0.773 ± 0.035	81.057 ± 0.009	18.943 ± 0.002
	SSGNet^38^	78.772 ± 0.081	83.784 ± 0.057	84.448 ± 0.082	0.768 ± 0.035	84.116 ± 0.068	15.884 ± 0.009
	FixMatch-LS^39^	73.327 ± 0.025	76.101 ± 0.017	77.700 ± 0.053	0.756 ± 0.029	76.900 ± 0.069	23.100 ± 0.058
	NCPLSL^40^	79.283 ± 0.008	82.364 ± 0.057	82.922 ± 0.059	0.781 ± 0.080	82.643 ± 0.083	17.357 ± 0.080
	FaxMatch^13^	80.937 ± 0.056	81.260 ± 0.085	82.837 ± 0.072	0.797 ± 0.065	82.048 ± 0.085	17.952 ± 0.046
	GANA-SFE^41^	78.684 ± 0.028	81.046 ± 0.086	83.614 ± 0.044	0.801 ± 0.088	82.330 ± 0.093	17.670 ± 0.016
	STFL^42^	77.402 ± 0.093	82.131 ± 0.039	83.671 ± 0.013	0.807 ± 0.023	82.901 ± 0.013	17.099 ± 0.059
	DL-AMC^44^	65.635 ± 0.057	70.587 ± 0.094	71.344 ± 0.080	0.746 ± 0.034	70.965 ± 0.003	29.035 ± 0.071
	ODLA-Net^45^	67.349 ± 0.015	71.259 ± 0.008	72.033 ± 0.066	0.753 ± 0.094	71.646 ± 0.047	28.354 ± 0.094
	VGG-16-GAN^46^	79.044 ± 0.052	81.003 ± 0.092	82.685 ± 0.017	0.789 ± 0.040	81.730 ± 0.050	18.270 ± 0.025
	GANViT-MD^47^	74.767 ± 0.015	75.575 ± 0.024	76.054 ± 0.030	0.775 ± 0.097	75.815 ± 0.051	24.185 ± 0.072
	PPO-GAN-MC^48^	82.321 ± 0.096	84.160 ± 0.099	85.628 ± 0.095	0.792 ± 0.082	84.894 ± 0.097	15.106 ± 0.050
Transfer learning	HE-TLMC^51^	80.877 ± 0.042	84.562 ± 0.055	85.005 ± 0.021	0.811 ± 0.037	84.784 ± 0.034	15.216 ± 0.052
	DLCA-SC^55^	82.564 ± 0.089	85.485 ± 0.046	86.929 ± 0.008	0.813 ± 0.084	86.207 ± 0.012	13.793 ± 0.022
	QDLM-NM^56^	72.524 ± 0.033	76.040 ± 0.017	76.725 ± 0.071	0.794 ± 0.031	76.382 ± 0.083	23.618 ± 0.037
	WE-TLMC^59^	69.652 ± 0.019	73.645 ± 0.015	74.385 ± 0.045	0.772 ± 0.027	74.015 ± 0.038	25.985 ± 0.100
	XAI-MRA^61^	79.769 ± 0.077	82.047 ± 0.086	84.484 ± 0.063	0.802 ± 0.059	83.266 ± 0.099	16.734 ± 0.006
	TL-MD^62^	70.934 ± 0.049	75.384 ± 0.036	76.088 ± 0.067	0.783 ± 0.005	75.736 ± 0.042	24.264 ± 0.002
Proposed	Proposed	89.537 ± 0.052	90.629 ± 0.032	91.325 ± 0.053	0.866 ± 0.100	90.977 ± 0.029	9.023 ± 0.016

**Table 8:** Performance comparison of ML, DL, TL, ablation, and proposed models on the PH2 dataset.

Category	Model name	Accuracy	F-measure	G-means	AUC	TPR	FNR
	MPMRF^24^	63.218 ± 0.092	67.754 ± 0.007	69.469 ± 0.032	0.699 ± 0.070	69.410 ± 0.016	30.590 ± 0.041
Machine leaning	BASCA-Opt^25^	66.109 ± 0.017	71.292 ± 0.026	72.203 ± 0.004	0.725 ± 0.073	71.652 ± 0.077	28.348 ± 0.088
	CFBD-CM^26^	63.538 ± 0.073	68.915 ± 0.014	70.184 ± 0.048	0.724 ± 0.019	69.839 ± 0.056	30.161 ± 0.038
	NIHSC-System^27^	64.383 ± 0.036	70.082 ± 0.012	70.390 ± 0.039	0.727 ± 0.065	70.189 ± 0.087	29.811 ± 0.027
	KNN-HFIP^28^	65.151 ± 0.040	72.058 ± 0.056	71.222 ± 0.055	0.737 ± 0.053	71.497 ± 0.062	28.503 ± 0.004
Deep learning	WDNR^6^	64.276 ± 0.038	70.686 ± 0.012	72.184 ± 0.023	0.748 ± 0.028	71.429 ± 0.002	28.571 ± 0.002
	ECSD-Net^29^	65.932 ± 0.026	71.448 ± 0.014	71.922 ± 0.085	0.749 ± 0.030	71.830 ± 0.009	28.170 ± 0.080
	DDCNN-F^33^	75.999 ± 0.044	80.984 ± 0.079	81.451 ± 0.068	0.710 ± 0.089	81.199 ± 0.044	18.801 ± 0.099
	YOLOv7-XAI^34^	76.543 ± 0.017	82.339 ± 0.003	84.763 ± 0.071	0.757 ± 0.089	83.543 ± 0.017	16.457 ± 0.068
	MRFO-SCC^37^	82.461 ± 0.027	82.838 ± 0.076	82.993 ± 0.076	0.783 ± 0.051	82.985 ± 0.091	17.015 ± 0.046
	SSGNet^38^	80.234 ± 0.026	85.322 ± 0.057	86.799 ± 0.055	0.788 ± 0.068	85.913 ± 0.033	14.087 ± 0.059
	FixMatch-LS^39^	75.038 ± 0.062	77.830 ± 0.065	79.100 ± 0.027	0.772 ± 0.023	78.825 ± 0.002	21.175 ± 0.047
	NCPLSL^40^	81.148 ± 0.027	83.925 ± 0.019	84.808 ± 0.041	0.802 ± 0.027	84.160 ± 0.001	15.840 ± 0.023
	FaxMatch^13^	83.204 ± 0.024	83.304 ± 0.036	84.461 ± 0.034	0.812 ± 0.001	84.450 ± 0.018	15.550 ± 0.040
	GANA-SFE^41^	80.279 ± 0.091	83.415 ± 0.041	85.996 ± 0.061	0.815 ± 0.027	83.482 ± 0.061	16.518 ± 0.041
	STFL^42^	79.125 ± 0.090	84.248 ± 0.046	85.718 ± 0.052	0.830 ± 0.062	85.135 ± 0.019	14.865 ± 0.079
	DL-AMC^44^	67.447 ± 0.064	71.890 ± 0.098	72.799 ± 0.096	0.757 ± 0.095	72.489 ± 0.036	27.511 ± 0.039
	ODLA-Net^45^	69.221 ± 0.044	72.822 ± 0.089	73.746 ± 0.079	0.773 ± 0.088	73.458 ± 0.044	26.542 ± 0.060
	VGG-16-GAN^46^	81.783 ± 0.047	83.259 ± 0.093	84.844 ± 0.008	0.793 ± 0.002	83.231 ± 0.089	16.769 ± 0.047
	GANViT-MD^47^	76.435 ± 0.065	77.418 ± 0.089	78.080 ± 0.055	0.790 ± 0.022	77.498 ± 0.064	22.502 ± 0.012
	PPO-GAN-MC^48^	84.234 ± 0.052	86.057 ± 0.065	88.152 ± 0.090	0.808 ± 0.086	88.023 ± 0.023	11.977 ± 0.032
Transfer learning	HE-TLMC^51^	82.599 ± 0.014	86.923 ± 0.029	86.727 ± 0.010	0.824 ± 0.073	86.887 ± 0.001	13.113 ± 0.048
	DLCA-SC^55^	84.971 ± 0.044	87.852 ± 0.095	89.413 ± 0.038	0.830 ± 0.044	88.824 ± 0.014	11.176 ± 0.021
	QDLM-NM^56^	73.901 ± 0.042	77.952 ± 0.001	78.964 ± 0.067	0.807 ± 0.072	78.177 ± 0.073	21.823 ± 0.049
	WE-TLMC^59^	71.684 ± 0.099	75.278 ± 0.074	76.539 ± 0.094	0.780 ± 0.099	75.665 ± 0.029	24.335 ± 0.020
	XAI-MRA^61^	81.847 ± 0.032	83.745 ± 0.044	86.633 ± 0.088	0.814 ± 0.003	85.323 ± 0.015	14.677 ± 0.072
	TL-MD^62^	72.647 ± 0.046	76.803 ± 0.021	78.019 ± 0.023	0.791 ± 0.056	77.310 ± 0.098	22.690 ± 0.054
Proposed	Proposed	91.140 ± 0.077	92.617 ± 0.016	93.383 ± 0.020	0.892 ± 0.060	92.740 ± 0.048	7.260 ± 0.063

#### Analysis of generalizability

We evaluated the generalizability of the proposed model by conducting experiments on the PH2 and DermNet datasets. These datasets provided additional context to assess their performance compared to existing models. Importantly, all samples from both PH2 and DermNet were used exclusively for evaluation purposes. No additional training or fine-tuning was performed on these datasets. Instead, we directly applied the model trained solely on the ISIC-2020 dataset. This zero-shot evaluation protocol provides a rigorous and unbiased method for testing the generalization of the model across domains and skin-type distributions, thereby avoiding dataset-specific adaptation.

The results are presented in Tables 7 and 8 for the PH2 and DermNet datasets. As seen in Table 7, the proposed model significantly outperforms all compared ML, DL, TL, and SSL methods on the PH2 dataset. The proposed method improves the F-measure by + 24.9%, G-means by + 29.8%, and TPR by + 21.9% compared to BASCA-Opt. Even compared to strong DL models like SSGNet and FaxMatch, F-measure improves by + 6.8% and + 11.5%. Compared to the best TL model (DLCA-SC), it achieves a 5.9% increase in F-measure, a 4.3% increase in G-means, and a 4.9% increase in TPR. These results highlight the robustness of the learned features beyond the distribution of the training data.

Similarly, Table 7 confirms the consistent superiority of our model on the DermNet dataset. The proposed model surpasses the best classical ML model (BASCA-Opt) by + 21.3% in F-measure, + 21.8% in G-means, and + 21.3% in TPR. Against strong SSL models, such as FixMatch-LS, NCPLSL, and GANA-SFE, the proposed SS-GAN achieves F-measure improvements of + 14.7% to + 18.6% and TPR improvements of + 14.2% to + 15.9%. Compared to the best TL model (DLCA-SC), the proposed model achieves a 5.4% increase in F-measure, a 4.4% increase in G-means, and a 4.4% increase in TPR.

These results show that the proposed SS-GAN performs exceptionally well across multiple external datasets without retraining. This confirms its strong generalization ability, robust feature extraction, and domain adaptability, which are key requirements for real-world dermatological diagnostic applications.

We performed two-tailed paired t-tests on six metrics (Accuracy, F-measure, G-means, AUC, TPR, FNR) to compare the proposed SS-GAN with top models on PH2 and DermNet datasets. On PH2, SS-GAN outperformed BASCA-Opt with p-values ranging from 0.0004 to 0.0009. Against PPO-GAN-MC, p-values ranged from 0.0012 to 0.0021. For DLCA-SC, values ranged from 0.0017 to 0.0028. On DermNet, SS-GAN showed stronger results compared to BASCA-Opt, with p-values ranging from 0.0002 to 0.0006. Against PPO-GAN-MC, p-values ranged from 0.0011 to 0.0017. For DLCA-SC, values ranged from 0.0019 to 0.0027. Overall, comparisons with state-of-the-art models on PH2 and DermNet show similarly low p-values. Confidence intervals (95%) were ± 0.01–0.08. These findings demonstrate that the proposed model surpasses existing models with high statistical significance. They confirm that the observed improvements are authentic and meaningful.

**Table 9:** Performance comparison of various GANs and the proposed SS-GAN models on the ISIC-2020 dataset.

Model	MMD	KLD	WD	MS
StyleGAN3	4.511 ± 0.003	5.202 ± 0.021	6.929 ± 0.031	0.531 ± 0.092
DRAGAN	8.224 ± 0.068	8.962 ± 0.072	9.565 ± 0.001	0.269 ± 0.027
AGE	7.821 ± 0.069	8.318 ± 0.094	9.029 ± 0.035	0.286 ± 0.071
α-GAN	7.238 ± 0.076	8.056 ± 0.087	8.653 ± 0.050	0.396 ± 0.028
DGAN	4.706 ± 0.075	5.108 ± 0.069	6.769 ± 0.073	0.528 ± 0.089
GAN	8.765 ± 0.037	9.809 ± 0.052	10.461 ± 0.076	0.199 ± 0.041
MELIIGAN	2.053 ± 0.075	3.626 ± 0.000	4.317 ± 0.095	0.682 ± 0.052
SRGAN	6.576 ± 0.010	7.620 ± 0.062	8.255 ± 0.079	0.402 ± 0.080
ESRGAN	4.125 ± 0.074	5.447 ± 0.039	6.121 ± 0.028	0.482 ± 0.024
StarSRGAN	5.255 ± 0.093	6.541 ± 0.016	7.184 ± 0.085	0.426 ± 0.002
SS-GAN	3.102 ± 0.053	4.985 ± 0.065	5.575 ± 0.007	0.572 ± 0.062
Proposed SS-GAN	0.264 ± 0.021	0.855 ± 0.087	0.877 ± 0.076	0.827 ± 0.097

**Table 10:** Performance comparison of various GANs and the proposed SS-GAN models on the HAM10000 dataset.

Model	MMD	KLD	WD	MS
StyleGAN3	3.511 ± 0.094	4.202 ± 0.019	5.929 ± 0.017	0.652 ± 0.081
DRAGAN	7.010 ± 0.088	7.904 ± 0.063	5.623 ± 0.055	0.360 ± 0.068
AGE	5.977 ± 0.067	7.089 ± 0.016	8.821 ± 0.086	0.466 ± 0.090
α-GAN	5.508 ± 0.014	6.267 ± 0.038	7.016 ± 0.001	0.474 ± 0.082
DGAN	3.849 ± 0.088	4.423 ± 0.068	5.219 ± 0.060	0.691 ± 0.088
GAN	7.731 ± 0.034	8.321 ± 0.045	9.124 ± 0.063	0.299 ± 0.032
MELIIGAN	1.514 ± 0.069	2.865 ± 0.044	3.639 ± 0.095	0.715 ± 0.023
SRGAN	5.129 ± 0.019	6.859 ± 0.048	7.677 ± 0.001	0.525 ± 0.090
ESRGAN	3.510 ± 0.032	4.629 ± 0.076	5.423 ± 0.044	0.543 ± 0.058
StarSRGAN	4.204 ± 0.083	5.462 ± 0.070	6.238 ± 0.031	0.558 ± 0.009
SS-GAN	2.569 ± 0.035	3.886 ± 0.018	4.674 ± 0.008	0.667 ± 0.087
Proposed SS-GAN	0.178 ± 0.027	0.602 ± 0.043	0.635 ± 0.011	0.923 ± 0.065

#### Analysis of SS-GAN

This section compares the proposed SS-GAN with various GAN variants selected for their relevance and effectiveness in medical imaging or generative tasks. These include style-based GAN version 3 (StyleGAN3)^71^ deep regret analytic GAN (DRAGAN)^72^ adversarial generator-encoder (AGE)^73^ alpha GAN (α-GAN)^74^ GAN, diffusion GAN (DGAN)^75^ melanoma high-fidelity GAN (MELIIGAN)^50^ super-resolution GAN (SRGAN)^76^ enhanced super-resolution GAN (ESRGAN)^77^ efficient-GAN (EGAN)^78^ StarSRGAN^79^ and the original SS-GAN. StyleGAN3 is well-known for generating high-quality, diverse image features, making it a benchmark in medical image synthesis. DRAGAN and AGE improve training stability and help prevent mode collapse, which is critical for medical datasets with class imbalance. α-GAN and DGAN are considered advanced hybrid GAN architectures that combine multiple generative and discriminative strategies. SRGAN, ESRGAN, and StarSRGAN have shown strong performance in medical image super-resolution. MELIIGAN is a recent GAN tailored for melanoma image fidelity, making it highly relevant for our domain.

We evaluate the performance of the proposed SS-GAN using four metrics: Maximum Mean Discrepancy (MMD), Kullback–Leibler Divergence (KLD), Wasserstein Distance (WD), and Mode Score (MS). These metrics assess diversity (mode coverage) and fidelity (realism) of generated samples. Both aspects are crucial for understanding mode collapse. MMD measures the distance between real and generated data distributions in a reproducing kernel Hilbert space. A lower MMD indicates better alignment and improved diversity. KLD quantifies divergence from the real distribution and is sensitive to missing modes. WD measures geometric distance between distributions, providing meaningful gradients and insights into fidelity. MS combines the number of distinct modes with discriminator confidence. It evaluates how well the generator produces varied yet realistic outputs.

To ensure fairness, all other model components were standardized across tests. The performance outcomes are detailed in Tables 9 and 10 for the ISIC-2020 and HAM10000 datasets. The proposed SS-GAN shows substantial improvements in both fidelity and diversity compared to leading GAN models, particularly StyleGAN3, MELIIGAN, and the baseline SS-GAN. On the ISIC-2020 dataset, it achieves an MMD of 0.264. This is 87% lower than MELIIGAN (2.053) and 94% lower than StyleGAN3 (4.511), indicating superior alignment with real data distributions. The KLD of 0.855 is 76% lower than MELIIGAN (3.626) and 84% lower than StyleGAN3 (5.202). Similarly, the WD of 0.877 is 79% lower than MELIIGAN (4.317) and 87% lower than StyleGAN3 (6.929), confirming improved realism. For diversity, the MS reaches 0.827, which is 21% higher than MELIIGAN (0.682) and over 55% better than SS-GAN (0.531). Similar trends are observed on HAM10000, with a 65% reduction in MMD and a 79% lower KLD compared to MELIIGAN, as well as nearly a 90% improvement over StyleGAN3. These results confirm the superior performance of SS-GAN.

We performed two-tailed paired t-tests on four GAN-specific metrics (MMD, KLD, WD, and MS) to evaluate the statistical significance of the proposed SS-GAN compared to other GAN models. On ISIC-2020, SS-GAN significantly outperforms MELIIGAN with p-values of 0.0006 (MMD), 0.0008 (KLD), 0.0004 (WD), and 0.0010 (MS). Comparisons with StyleGAN3 yield even lower p-values: 0.0003, 0.0005, 0.0002, and 0.0009, confirming the strong improvements. Confidence intervals are narrow (± 0.01 to ± 0.03), validating the findings at a 95% confidence level. On HAM10000, SS-GAN again surpasses MELIIGAN with p-values of 0.0004, 0.0007, 0.0005, and 0.0009, and StyleGAN3 with 0.0002, 0.0004, 0.0003, and 0.0007. Confidence intervals remain tight (± 0.01 to ± 0.04) at the same confidence level. Overall, comparisons with state-of-the-art GAN architectures on both datasets yield low p-values and narrow confidence bounds. These results reinforce the reliability of our enhancements and confirm that the proposed SS-GAN generates diverse, realistic, and generalizable melanoma samples.

**Fig. 11:**
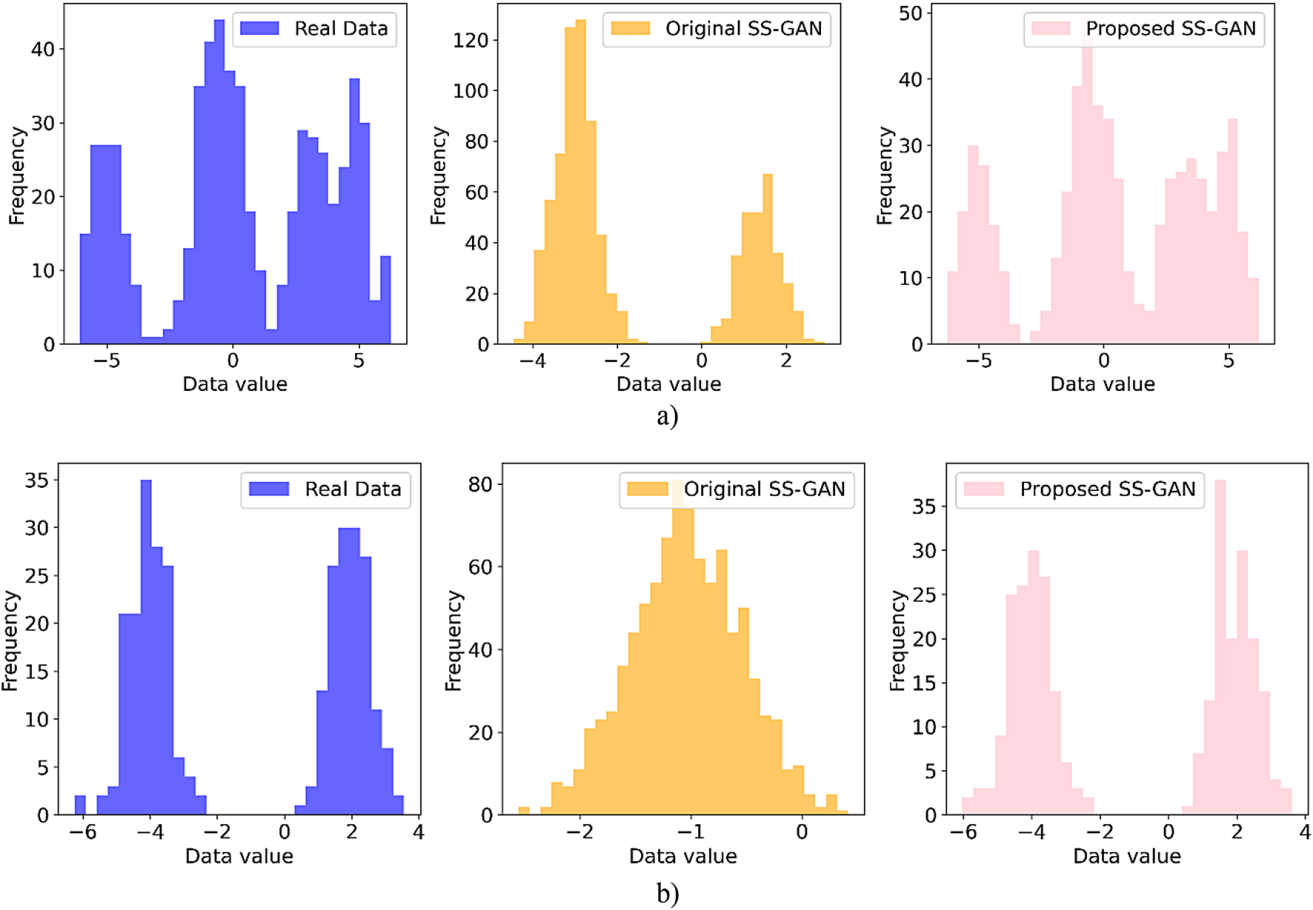
Real vs. generated data distributions for original and proposed SS-GAN models on the (**a**) ISIC-2020 and (**b**) HAM10000 datasets.

Figure 10 compares the data distributions of the original and proposed SS-GAN with real data for the ISIC-2020 and HAM10000 datasets. The proposed SS-GAN closely aligns with the real data distribution, effectively avoiding the mode collapse seen in the original SS-GAN, which concentrates around fewer data modes. This improvement comes from integrating reconstruction loss, self-attention mechanisms, and consistency regularization. These components enhance feature diversity and stabilize the training process. As a result, the proposed model captures a wider range of data variations and preserves the multimodal structure. This ensures the generation of realistic and representative samples compared to conventional GAN models.

Figure 11 visualizes feature vectors generated by the model generator for the ISIC-2020 and HAM10000 datasets. These outputs are originally high-dimensional. Direct visualization of these high-dimensional vectors is difficult, so PCA was applied to reduce them to two dimensions for clearer interpretation. The left plot illustrates the distribution of generated features with reconstruction loss, showing wide dispersion that reflects improved diversity and reduced mode collapse. In contrast, the right plot (without reconstruction loss) reveals dense and localized clusters, indicating limited variability. This comparison confirms that reconstruction loss mitigates mode collapse, allowing the generator to create richer feature representations, which are essential for robust melanoma classification.

**Fig. 12:**
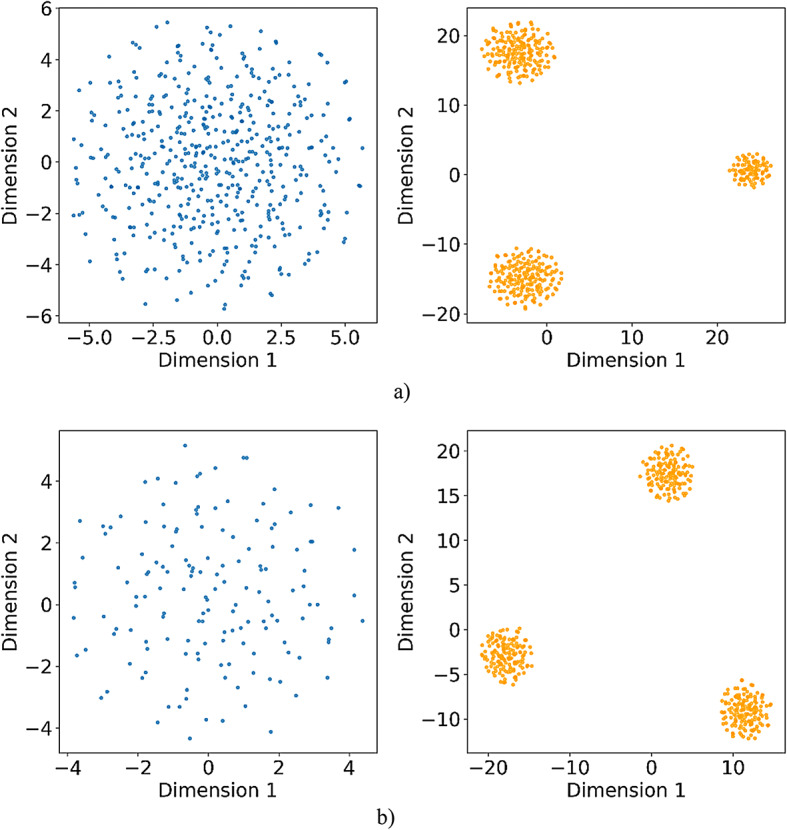
PCA-based visualization of feature vectors generated by the model generator for (**a**) ISIC-2020 and (**b**) HAM10000 datasets. The right plot (without reconstruction loss) displays dense clustering caused by mode collapse, whereas the left plot (with reconstruction loss) demonstrates improved feature diversity and distribution.

**Table 11:** Comparative performance of basic and metaheuristic optimization methods for hyperparameter optimization on the ISIC-2020 dataset.

Algorithm type	Method	Accuracy	F-measure	G-means	AUC	TPR	FNR
Basic	RS	68.511 ± 0.013	70.202 ± 0.061	71.929 ± 0.035	0.655 ± 0.095	70.699 ± 0.148	29.301 ± 0.099
	GS	78.164 ± 0.099	79.877 ± 0.015	80.561 ± 0.099	0.781 ± 0.021	80.040 ± 0.058	19.960 ± 0.097
	BO	84.082 ± 0.073	85.248 ± 0.077	86.913 ± 0.039	0.815 ± 0.064	85.741 ± 0.042	14.259 ± 0.018
	HB	80.478 ± 0.007	82.449 ± 0.013	83.091 ± 0.082	0.798 ± 0.010	82.889 ± 0.014	17.111 ± 0.072
Evolutionary	HMS	70.989 ± 0.087	72.485 ± 0.083	74.158 ± 0.074	0.679 ± 0.087	72.664 ± 0.035	27.336 ± 0.038
	SSA	71.309 ± 0.096	73.723 ± 0.007	74.342 ± 0.073	0.699 ± 0.091	74.185 ± 0.056	25.815 ± 0.032
	COA	72.969 ± 0.082	74.161 ± 0.013	76.790 ± 0.011	0.704 ± 0.091	74.408 ± 0.082	25.592 ± 0.074
	FA	73.434 ± 0.091	75.187 ± 0.038	76.818 ± 0.066	0.713 ± 0.030	76.802 ± 0.046	23.198 ± 0.047
	BA	74.362 ± 0.016	75.777 ± 0.007	76.966 ± 0.085	0.745 ± 0.091	76.298 ± 0.042	23.702 ± 0.063
	PSO	75.192 ± 0.066	76.918 ± 0.025	77.549 ± 0.000	0.749 ± 0.100	77.032 ± 0.034	22.968 ± 0.089
	DE	75.409 ± 0.016	77.709 ± 0.092	78.344 ± 0.031	0.753 ± 0.017	78.276 ± 0.046	21.724 ± 0.083
	CMA-ES	78.421 ± 0.099	79.650 ± 0.088	80.304 ± 0.049	0.785 ± 0.018	79.910 ± 0.094	20.090 ± 0.056
	PO	79.708 ± 0.017	80.900 ± 0.060	81.591 ± 0.021	0.792 ± 0.030	80.906 ± 0.056	19.094 ± 0.033
	AOA	80.384 ± 0.025	82.144 ± 0.064	83.793 ± 0.021	0.802 ± 0.031	82.895 ± 0.098	17.105 ± 0.071
	ABC	76.183 ± 0.018	77.899 ± 0.014	79.395 ± 0.010	0.768 ± 0.027	78.98 ± 0.0942	21.020 ± 0.06.
Proposed	ML-ABC	90.517 ± 0.005	92.769 ± 0.021	93.508 ± 0.016	0.893 ± 0.026	92.825 ± 0.027	7.175 ± 0.061

**Table 12:** Comparative performance of basic and metaheuristic optimization methods for hyperparameter optimization on the HAM10000 dataset.

Algorithm type	Method	Accuracy	F-measure	G-means	AUC	TPR	FNR
Basic	RS	71.511 ± 0.077	72.202 ± 0.046	72.929 ± 0.046	0.682 ± 0.032	72.451 ± 0.042	27.549 ± 0.038
	GS	80.359 ± 0.082	81.212 ± 0.018	82.969 ± 0.056	0.792 ± 0.033	82.737 ± 0.044	17.263 ± 0.096
	BO	86.636 ± 0.018	86.077 ± 0.023	87.892 ± 0.044	0.824 ± 0.027	86.713 ± 0.084	13.287 ± 0.013
	HB	82.009 ± 0.089	83.227 ± 0.083	84.010 ± 0.055	0.801 ± 0.056	83.317 ± 0.01	16.683 ± 0.011
Evolutionary	HMS	72.620 ± 0.084	74.897 ± 0.089	75.672 ± 0.049	0.688 ± 0.044	75.094 ± 0.067	24.906 ± 0.074
	SSA	73.243 ± 0.056	74.100 ± 0.005	75.894 ± 0.075	0.704 ± 0.097	74.571 ± 0.037	25.429 ± 0.054
	COA	74.886 ± 0.052	76.244 ± 0.027	77.795 ± 0.042	0.716 ± 0.058	77.398 ± 0.078	22.602 ± 0.076
	FA	75.711 ± 0.025	76.713 ± 0.010	77.807 ± 0.041	0.719 ± 0.068	77.375 ± 0.053	22.625 ± 0.061
	BA	76.474 ± 0.040	78.255 ± 0.042	79.831 ± 0.089	0.751 ± 0.087	79.083 ± 0.02	20.917 ± 0.076
	PSO	76.692 ± 0.072	78.879 ± 0.063	79.965 ± 0.011	0.758 ± 0.051	79.338 ± 0.099	20.662 ± 0.124
	DE	77.498 ± 0.038	79.811 ± 0.049	80.710 ± 0.037	0.761 ± 0.091	80.149 ± 0.045	19.851 ± 0.077
	CMA-ES	79.870 ± 0.049	81.027 ± 0.018	82.809 ± 0.042	0.791 ± 0.049	81.44 ± 0.018	18.560 ± 0.069
	PO	80.791 ± 0.081	81.984 ± 0.097	83.766 ± 0.015	0.798 ± 0.029	82.551 ± 0.086	17.449 ± 0.032
	AOA	82.187 ± 0.003	83.814 ± 0.016	84.574 ± 0.064	0.815 ± 0.011	84.206 ± 0.035	15.794 ± 0.095
	ABC	78.219 ± 0.088	80.384 ± 0.058	81.217 ± 0.064	0.772 ± 0.072	80.768 ± 0.013	19.232 ± 0.029
Proposed	ML-ABC	92.358 ± 0.086	93.376 ± 0.074	94.500 ± 0.051	0.902 ± 0.020	93.482 ± 0.049	6.518 ± 0.032

#### Analysis of the proposed ML-ABC

This section compares the performance of ML-ABC with several popular hyperparameter tuning methods. The analysis considers three basic methods: random search (RS), grid search (GS), Bayesian optimization (BO), and Hyperband (HB). It also evaluates eleven evolutionary algorithms, including human mental search (HMS), salp swarm algorithm (SSA), cuckoo optimization algorithm (COA), firefly algorithm (FA), bat algorithm (BA), particle swarm optimization (PSO), deferential evolution (DE), CMA-ES, puma optimizer (PO)^80^ arithmetic optimization algorithm (AOA)^81^ and the original ABC.

To ensure fair assessment, uniformity was maintained across all model variables during evaluations. The results from the ISIC-2020 and HAM10000 datasets are detailed in Tables 10 and 11. Compared to BO, ML-ABC improves accuracy by 7.6% on ISIC-2020 (90.52% vs. 84.08%) and 6.6% on HAM10000 (92.36% vs. 86.63%). Against the top evolutionary algorithm, AOA, ML-ABC shows gains of 10% on ISIC-2020 (90.52% vs. 80.38%) and 12% on HAM10000 (92.36% vs. 82.18%). Compared to the original ABC, ML-ABC boosts accuracy by 18.9% on ISIC-2020 and 18.1% on HAM10000. F-measure and G-means show similar improvements. These gains result from the ability of ML-ABC to share knowledge among sub-populations, accelerate convergence, and avoid local optima. Overall, ML-ABC demonstrates superior stability and generalization compared to all baselines.

We conducted two-tailed paired t-tests on six metrics (Accuracy, F-measure, G-means, AUC, TPR, FNR) to establish statistical significance. The proposed ML-ABC was compared with BO, the strongest basic optimizer, and the AOA, the leading evolutionary optimizer. On the ISIC-2020 dataset, ML-ABC outperformed BO with p-values ranging from 0.0015 to 0.0023 across all metrics. Comparisons with AOA showed p-values of less than 0.003. A similar trend was observed on the HAM10000 dataset, where p-values ranged from 0.0015 to 0.0024 against BO and remained below 0.0035 against AOA, confirming robustness. Confidence intervals for ML-ABC were narrow (± 0.02–±0.04 at a 95% confidence level), confirming its stability and reliability. Overall, each comparison shows that ML-ABC achieves faster convergence, robust exploration, and superior performance compared to other optimization techniques.

**Fig. 13:**
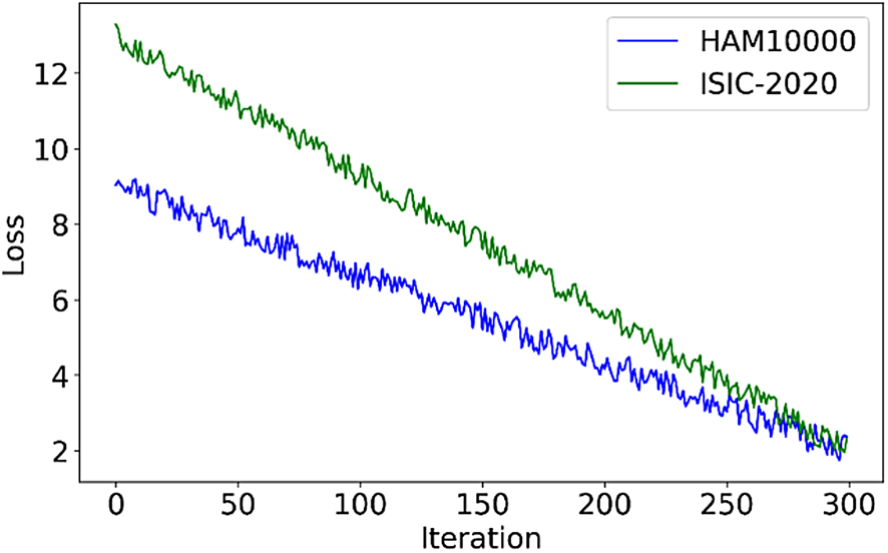
Loss minimization curves over 300 iterations for ISIC-2020 and HAM10000 using the ML-ABC method.

**Fig. 14:**
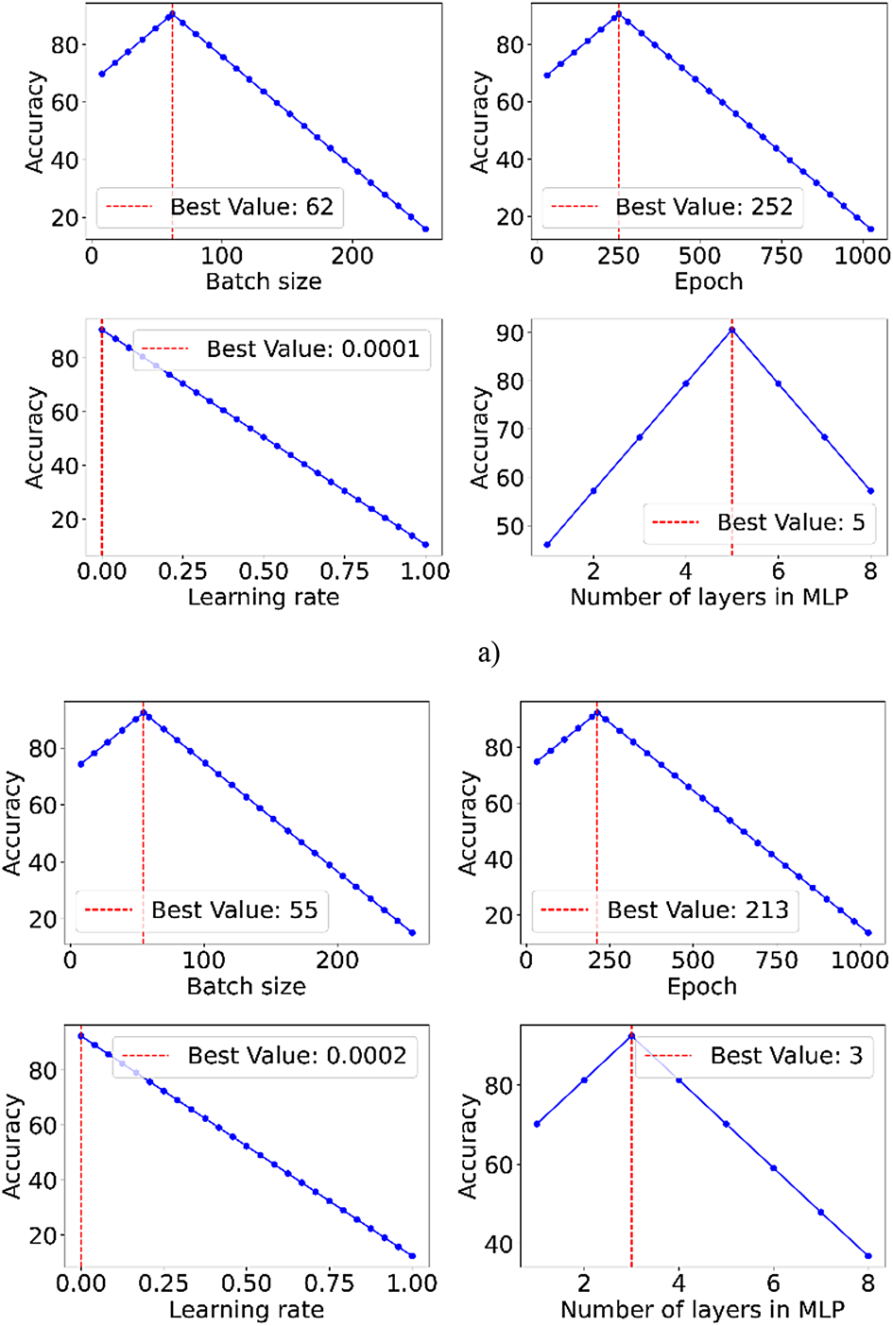
Loss minimization curves over 300 iterations for ISIC-2020 and HAM10000 using the ML-ABC method.

Figure 13 shows the loss minimization process over 300 iterations for ISIC-2020 and HAM10000 using the proposed ML-ABC method. Both curves display a smooth and steady decline, confirming the strong convergence capability of the algorithm. The HAM10000 dataset exhibits a slightly faster reduction in loss, while ISIC-2020 shows consistent stability across iterations. This result demonstrates the effectiveness of the mutual learning component in ML-ABC, which improves convergence and enhances hyperparameter tuning.

Figure 14 illustrates the effectiveness of the ML-ABC algorithm in optimizing key generator hyperparameters for the ISIC-2020 and HAM10000 datasets. The figure indicates that the algorithm identifies optimal values for batch size, number of epochs, learning rate, and MLP layers, achieving maximum accuracy for each dataset. Notably, the curves display clear peaks at the optimal configurations, confirming the sensitivity of the model to tuning. These results demonstrate that ML-ABC effectively explores the search space, avoids local optima, and identifies hyperparameter settings that maximize performance.

## Discussion

The proposed model enhances an SS-GAN using an ML-ABC algorithm for hyperparameter tuning. It introduces reconstruction loss in the generator to reduce mode collapse. It also incorporates self-attention into both the generator and discriminator to capture long-range dependencies and enhance feature extraction. CR stabilizes predictions under data augmentations. Pseudo-labeling includes only high-confidence unlabeled samples to improve label quality. Together, CR and pseudo-labeling help address class imbalance. CR improves generalization for minority classes, while pseudo-labeling generates reliable labels for underrepresented samples. Reconstruction loss encourages feature diversity. Self-attention helps focus on lesion areas that traditional models might miss. ML-ABC handles hyperparameter tuning. This algorithm combines global exploration with local exploitation, supporting knowledge sharing between candidate solutions. This strategy accelerates convergence, avoids local optima, and leads to better model configurations. Compared to conventional ABC or other metaheuristics, the model shows improved accuracy, stability, and reproducibility. This combination of architectural improvements and optimization makes the model robust and efficient. It performs well in classifying dermoscopic images, even when the labeled data are limited.

Through these innovations, the model outperforms state-of-the-art methods on ISIC-2020, HAM10000, PH2, and DermNet datasets. It achieves superior performance by enhancing generalization, handling imbalanced data, and effectively utilizing both labeled and unlabeled samples. These findings empirically validate all five hypotheses proposed in Sections1–2. The ablation studies demonstrate that each architectural enhancement, including reconstruction loss, self-attention, consistency regularization, confidence-based pseudo-labeling, and improved ABC optimization, makes a unique contribution to the final performance. Together, they form a synergistic framework that significantly enhances generalization, robustness, and diagnostic accuracy across datasets. This confirms that the model effectively addresses challenges such as mode collapse, feature extraction, label noise, and hyperparameter tuning across various clinical conditions.

Beyond technical performance, it is crucial to consider the clinical implications, particularly for melanoma detection, where diagnostic accuracy can have life-critical consequences. To align technical metrics with clinical needs, we emphasize sensitivity (TPR) and FNR. These are critical for melanoma detection, where a missed diagnosis can have severe consequences. As shown in Tables 4, 5 and 7, and 8, the proposed SS-GAN consistently achieves a TPR above 90%. It maintains an FNR below 10%, ensuring that the vast majority of melanoma cases are correctly identified. These results demonstrate that improvements in F-measure and G-means directly translate to clinically meaningful outcomes, reducing false negatives and enhancing diagnostic confidence. Compared to baseline models, the proposed SS-GAN decreases the FNR by up to 30%, significantly minimizing the risk of undetected melanoma. This alignment between metrics and real-world diagnostic needs underscores the potential of the framework for supporting clinical decision-making.

The proposed SS-GAN offers clear advantages over various GAN-based models, including StyleGAN3, DRAGAN, AGE, α-GAN, DGAN, MELIIGAN, SRGAN, ESRGAN, EGAN, and StarSRGAN. StyleGAN3 is known for visually impressive results, but it does not explicitly mitigate mode collapse. In contrast, SS-GAN includes a reconstruction loss that improves structural fidelity and feature diversity. DRAGAN and AGE improve training stability but lack self-attention and consistency regularization, which are essential for modeling long-range dependencies in dermoscopic images. α-GAN and DGAN, although based on hybrid and diffusion frameworks, do not address class imbalance or the utilization of unlabeled data. SS-GAN addresses these challenges by utilizing confidence-based pseudo-labeling and consistency regularization. MELIGAN, while melanoma-focused, prioritizes fidelity over diversity and does not address label scarcity. SS-GAN is more robust in semi-supervised melanoma contexts. SRGAN and ESRGAN target super-resolution, EGAN prioritizes speed, and StarSRGAN focuses on quality. However, none match SS-GAN in balancing diversity and fidelity, as shown by its better MMD, KLD, WD, and mode scores. Unlike many GANs, such as StyleGAN3 or MELIGAN, SS-GAN does not generate synthetic images. Instead, its generator creates high-dimensional feature vectors that mimic the distributions of real dermoscopic images. These are used only in training. All evaluations and clinical analyses rely exclusively on real images. Although SS-GAN incurs a higher computational cost due to self-attention and ML-ABC optimization, these elements ensure strong generalization and top-tier performance across datasets.

The theoretical and practical aspects of the work are significant. Theoretically, adding reconstruction loss, self-attention, and consistency regularization advances semi-supervised learning. These components reduce mode collapse and improve feature diversity. Pseudo-labeling extends the learning capacity by effectively using unlabeled data. ML-ABC introduces a rigorous optimization framework for hyperparameters, which is crucial for ensuring the stability of GANs. Practically, the model demonstrates robust performance across heterogeneous datasets, such as ISIC-2020 and HAM10000, thereby proving its generalization capability. Its ability to handle imbalanced datasets and noisy labels makes it ideal for clinical settings, where labeled data are often scarce. For real-time applications, the model can be deployed using pre-trained weights, requiring only lightweight inference on dermoscopic images. With GPU acceleration, it classifies melanoma and non-melanoma lesions within milliseconds. This speed makes it suitable for mobile diagnostic tools or clinical decision support systems that assist dermatologists in early melanoma detection.

The proposed model can be applied to domains where limited labeled data and class imbalance pose challenges. For example, in medical imaging tasks such as breast cancer detection using mammograms or lung nodule classification in computed tomography (CT) scans, the semi-supervised structure effectively utilizes unlabeled datasets. This improves accuracy. Similarly, reconstruction loss and self-attention capture fine-grained features and spatial dependencies. This makes the model suitable for histopathology image analysis, retinal disease detection, or magnetic resonance imaging (MRI)-based tumor segmentation. Outside healthcare, the approach can benefit industrial defect detection, satellite imagery analysis, and security applications where labeled data are costly to obtain. By adapting the generator and discriminator architectures to the specific domain and using ML-ABC for hyperparameter optimization, the model can deliver state-of-the-art performance. It ensures diversity and fidelity in feature representation while remaining computationally efficient.

### Key findings summary

The key outcomes of the comparative evaluation between the proposed SS-GAN model and the best state-of-the-art baselines across the ISIC-2020, HAM10000, PH2, and DermNet datasets are summarized as follows:

- Accuracy improvements:
- ISIC-2020: Increased from 87.86% (PPO-GAN-MC) to 90.52% (Proposed) → +3.03%.
- HAM10000: Increased from 89.24% (DLCA-SC) to 92.36% (Proposed) → +3.12%.
- PH2: Increased from 82.56% (DLCA-SC) to 89.54% (Proposed) → +6.97%.
- DermNet: Increased from 84.97% (DLCA-SC) to 91.14% (Proposed) → +6.17%.
- Precision improvements:
- ISIC-2020: Increased from 87.90% (PPO-GAN-MC) to 93.00% (Proposed) → +5.10%.
- HAM10000: Increased from 90.10% (DLCA-SC) to 93.60% (Proposed) → +3.50%.
- PH2: Increased from 85.20% (DLCA-SC) to 91.00% (Proposed) → +5.80%.
- DermNet: Increased from 87.50% (DLCA-SC) to 92.90% (Proposed) → +5.40%.
- F-measure improvements:
- ISIC-2020: Increased from 88.15% (PPO-GAN-MC) to 92.77% (Proposed) → +4.62%.
- HAM10000: Increased from 90.40% (DLCA-SC) to 93.38% (Proposed) → +2.98%.
- PH2: Increased from 85.49% (DLCA-SC) to 90.63% (Proposed) → +5.14%.
- DermNet: Increased from 87.85% (DLCA-SC) to 92.62% (Proposed) → +4.77%.
- G-means improvements:
- ISIC-2020: Increased from 89.97% (DLCA-SC) to 93.51% (Proposed) → +3.54%.
- HAM10000: Increased from 90.88% (DLCA-SC) to 94.50% (Proposed) → +3.62%.
- PH2: Increased from 86.93% (DLCA-SC) to 91.33% (Proposed) → +4.40%.
- DermNet: Increased from 89.41% (DLCA-SC) to 93.38% (Proposed) → +3.97%.
- AUC improvements:
- ISIC-2020: Increased from 0.845 (DLCA-SC) to 0.893 (Proposed) → +4.74% (relative).
- HAM10000: Increased from 0.858 (DLCA-SC) to 0.902 (Proposed) → +5.13% (relative).
- PH2: Increased from 0.813 (DLCA-SC) to 0.866 (Proposed) → +6.52% (relative).
- DermNet: Increased from 0.830 (DLCA-SC) to 0.892 (Proposed) → +7.47% (relative).
- TPR improvements:
- ISIC-2020: Increased from 88.78% (PPO-GAN-MC) to 92.83% (Proposed) → +4.05%.
- HAM10000: Increased from 90.55% (DLCA-SC) to 93.48% (Proposed) → +2.93%.
- PH2: Increased from 86.21% (DLCA-SC) to 90.98% (Proposed) → +4.77%.
- DermNet: Increased from 88.82% (DLCA-SC) to 92.74% (Proposed) → +3.92%.
- FNR reduction:
- ISIC-2020: Decreased from 11.22% (PPO-GAN-MC) to 7.17% (Proposed) → − 36.1%.
- HAM10000: Decreased from 9.45% (DLCA-SC) to 6.52% (Proposed) → − 30.9%.
- PH2: Decreased from 13.79% (DLCA-SC) to 9.02% (Proposed) → − 34.6%.
- DermNet: Decreased from 11.18% (DLCA-SC) to 7.26% (Proposed) → − 35.1%.

Ablation results confirmed the contributions of individual components:

- RL minimized mode collapse and maintained diverse feature representations.
- SA improved feature extraction by capturing long-range dependencies and focusing on critical lesion patterns.
- CR stabilized predictions and enhanced generalization on unlabeled data.
- PL enhanced label quality by leveraging high-confidence unlabeled samples.
- ML-ABC optimization achieved superior hyperparameter tuning compared to baseline methods, boosting classification accuracy.

These innovations helped the proposed model outperform state-of-the-art approaches in melanoma detection. The model maintained high robustness, especially under limited labeled data conditions.

### Limitations

The limitations of the proposed model and its potential solutions are as follows:

- Label noise sensitivity: The performance of SS-GAN, like many ML models, can be severely impacted by noise in labeled data. In medical datasets, diagnostic labels come from expert analyses. Even small errors in these labels can disrupt the learning process of the model. Such errors may cause the generator to produce misleading or incorrect samples, thereby reducing the overall model performance. To mitigate this, we propose incorporating label cleaning techniques such as automated label correction algorithms or robust outlier detection methods during preprocessing. Self-training, which iteratively labels high-confidence samples, and active learning, which queries informative samples for labeling, can also reduce label noise. Robust loss functions that down-weight noisy samples and bootstrapping methods can further improve the resilience of the model to label errors. Applying these solutions before or during training ensures the model learns from high-quality labels, improving performance and generalizability.
- Lack of clinical validation and expert feedback: The proposed SS-GAN shows strong quantitative performance on benchmark datasets. However, its clinical applicability has not yet been validated by dermatologists or in real-world diagnostic workflows. This may limit its acceptance in clinical environments where interpretability, trust, and reliability are critical. Future work should involve collaboration with dermatologists to conduct validation studies, run clinical trials, and compare outputs with expert evaluations. Clinician feedback can guide the fine-tuning of the model to ensure its decisions align with established medical practices. This will enhance trust and usability in real healthcare settings.
- Computational efficiency and resource constraints: The proposed SS-GAN model shows promising performance. However, its GPU usage and training time may hinder deployment on devices with limited processing power. The architecture, which incorporates components such as self-attention and ML-based hyperparameter optimization, requires substantial computational resources. This makes implementing low-cost clinical devices difficult. This could limit its practicality in real-time applications, especially in smaller clinics or resource-constrained settings. Future work should optimize the architecture of the model for efficiency. Techniques such as pruning, quantization, or lightweight variants can reduce resource usage without compromising performance. Cloud-based solutions for offloading heavy tasks could also enable real-time analysis without straining local hardware.
- Dataset diversity and demographic bias: Although widely accepted datasets, such as ISIC-2020 and HAM10000, are used, they underrepresent darker skin tones (Fitzpatrick types IV–VI). This imbalance may cause the model to perform better on lighter skin and reduce diagnostic reliability across ethnic groups. The PH2 dataset facilitates evaluation of generalizability due to its standardized imaging; however, it has a limited number of samples from a narrow demographic. The DermNet dataset introduces variation in imaging and lesion types, but it lacks demographic details such as skin tone and ethnicity. Therefore, any claims of generalizability remain limited. Future work should include datasets with balanced Fitzpatrick skin types and external validation on diverse cohorts for fairness and robustness.
- Pseudo-label reliability: Mechanisms like self-attention, confidence-based pseudo-labeling, and consistency regularization reduce incorrect label propagation, but do not fully eliminate it. SSL models remain sensitive to mislabeled pseudo-samples, which can introduce bias or degrade performance if they are reinforced during training. Our approach mitigates this risk by filtering low-confidence samples and stabilizing predictions, but a full guarantee of correctness is challenging. Future work could investigate noise-robust pseudo-labeling methods, such as curriculum learning, co-training, or ensemble-based label refinement, to enhance the reliability of pseudo-labels.

## Conclusion

This study introduced a semi-supervised learning framework for melanoma classification. It addresses key limitations of conventional SS-GANs, including mode collapse, weak global feature modeling, poor generalization, and unreliable pseudo-labeling. The proposed model integrates four key innovations. These include reconstruction loss to preserve diversity, self-attention for capturing long-range dependencies, consistency regularization to stabilize predictions, and confidence-guided pseudo-labeling to filter noisy supervision. Additionally, an enhanced ML-ABC algorithm with Random Key encoding was employed to optimize hyperparameters efficiently. Extensive evaluations on four benchmark datasets (ISIC-2020, HAM10000, PH2, and DermNet) demonstrated superior diagnostic accuracy. The proposed method achieved F-measures of above 90%, consistently outperforming state-of-the-art baselines. These results confirm strong generalization and stability with limited labeled data. They represent a significant advancement in melanoma detection through semi-supervised learning and evolutionary optimization.

Future work can explore several avenues to enhance further the capabilities of the proposed SS-GAN model for melanoma detection. First, integrating multimodal data sources, such as genetic markers, patient demographics, and historical health records, could be pursued. This approach enables the model to incorporate richer features that enhance diagnostic accuracy beyond visual patterns in dermatoscopic images. Using such diverse data inputs can improve understanding of melanoma characteristics, enabling earlier and more accurate diagnoses. We also plan to extend the SS-GAN model beyond binary classification to multi-class classification of lesion types in datasets like HAM10000. This will enable the model to distinguish between multiple skin disease categories, rather than just two classes. To achieve this, we will explore transfer learning methods to fine-tune the model on more lesion classes and other dermatology datasets. This expansion will improve the robustness and accuracy of the model, while significantly increasing its potential impact in dermatology.

## Data Availability

The datasets used and/or analysed during the current study available from the corresponding author on reasonable request.
